# Pulmonary embolism and deep venous thrombosis after COVID-19: long-term risk in a population-based cohort study

**DOI:** 10.1016/j.rpth.2023.100284

**Published:** 2023-06-21

**Authors:** Helen Sjöland, Martin Lindgren, Triantafyllia Toska, Per-Olof Hansson, Katarina Glise Sandblad, Christian Alex, Lena Björck, Ottmar Cronie, Jonas Björk, Christina E. Lundberg, Martin Adiels, Annika Rosengren

**Affiliations:** 1 Department of Molecular and Clinical Medicine, Institute of Medicine, Sahlgrenska Academy, University of Gothenburg, Gothenburg, Sweden; 2 Department of Medicine Geriatrics and Emergency Medicine, Sahlgrenska University Hospital, Östra Hospital, Region Västra Götaland, Gothenburg, Sweden; 3 School of Public Health and Community Medicine, Institute of Medicine, University of Gothenburg, Gothenburg, Sweden; 4 Division of Occupational and Environmental Medicine, Lund University, Lund, Sweden; 5 Clinical Studies Sweden, Forum South, Skåne University Hospital, Lund, Sweden; 6 Department of Food and Nutrition, and Sport Science, University of Gothenburg, Gothenburg, Sweden

**Keywords:** COVID-19/complications, COVID-19/epidemiology, pulmonary embolism, venous thromboembolism, venous thrombosis

## Abstract

**Background:**

Venous thromboembolism (VTE) (pulmonary embolism [PE] or deep venous thrombosis [DVT]) is common during acute COVID-19. Long-term excess risk has not yet been established.

**Objectives:**

To study long-term VTE risk after COVID-19.

**Methods:**

Swedish citizens aged 18 to 84 years hospitalized and/or testing positive for COVID-19 between January 1, 2020, and September 11, 2021 (exposed), stratified by initial hospitalization, were compared to matched (1:5), nonexposed, population-derived subjects without COVID-19. Outcomes were incident VTE, PE, or DVT recorded within 60, 60 to <180, and ≥180 days. Cox regression was used for evaluation, and a model adjusted for age, sex, comorbidities, and socioeconomic markers was developed to control for confounders.

**Results:**

Among exposed patients, 48,861 were hospitalized for COVID-19 (mean age, 60.6 years) and 894,121 were without hospitalization (mean age, 41.4 years). Among patients hospitalized for COVID-19, fully adjusted hazard ratios during 60 to <180 days were 6.05 (95% CI, 4.80-7.62) for PE and 3.97 (CI, 2.96-5.33) for DVT compared with that for nonexposed patients with corresponding estimates among those with COVID-19 without hospitalization 1.17 (CI, 1.01-1.35) and 0.99 (CI, 0.86-1.15), based on 475 and 2311 VTE events, respectively. Long-term (≥180 days) hazard ratios in patients hospitalized for COVID-19 were 2.01 (CI, 1.51-2.68) for PE and 1.46 (CI, 1.05-2.01) for DVT, while nonhospitalized patients had similar risk as nonexposed patients, based on 467 and 2030 VTE events, respectively.

**Conclusion:**

Patients hospitalized for COVID-19 retained an elevated excess risk of VTE, mainly PE, after 180 days, while long-term risk of VTE in individuals with COVID-19 without hospitalization was similar to that in the nonexposed patients.

## Essentials

• The long-term risk of venous thromboembolism (VTE) after COVID-19 is incompletely understood.
• We studied nationwide Swedish registers for long-term risk of VTE after COVID-19 (*n* = 5,655,635).
• In COVID-19 cases without hospitalization, the VTE risk had returned to baseline after 60 days.
• After hospitalization for COVID-19, risk of pulmonary embolism was still elevated after 180 days.

## 1 Introduction

In most people infected with SARS-CoV-2, the clinical course is relatively mild and without sequelae, but serious pulmonary complications requiring hospitalization, including intensive care, occur in a subset. Currently, almost 3 years after its first occurrence, complications during follow-up are being increasingly recognized.

Thromboembolic events, such as pulmonary embolism (PE) and deep venous thrombosis (DVT), were promptly recognized as common complications in severe COVID-19 [[1], [2], [3], [4], [5]]. The expected baseline rate of venous thromboembolism (VTE) is 1 to 3 cases per 1000 individuals per year, with a predominance of DVT over PE [6,7], and associated with a number of well-established risk factors such as older age [[8], [9], [10]]. Early evaluations rating PE as the most prevalent VTE in COVID-19 [1,2,5,11] were later contested in meta-analyses [4,12], but the distribution between PE and DVT has not yet been reliably described. Also, the increase in PE raised concerns about the risk of persistent symptoms, which may affect around half of PE survivors [13]. Increasing evidence demonstrated an association between the severity of acute COVID-19 and complications, indicating a potential long-time elevated risk of VTE in a considerable number of patients [14,15].

While the short-term high risk of VTE in connection with severe COVID-19 is well known, the extent to which elevated risk persists in nonsevere cases of infection has not yet been reliably established. A prior Swedish matched-cohort study, using the same registers available to us, found high risks of VTE in mild, nonhospitalized cases, although much higher among the severely affected [16], with persistent high rates 2 to 4 months after diagnosis. However, only a limited number of observations were available for long-term analyses. Also, VTE was included as part of a recent British cohort study of cardiometabolic outcomes up to 1 year after COVID-19, with similar results, although with VTE analyzed in conjunction with other cardiovascular diseases (CVDs) [17]. The research group analyzed a large primary care database, including both laboratory-confirmed and clinical diagnoses of COVID-19 but largely excluding patients with prior CVD and accordingly selecting a comparatively healthy cohort. Further, initially hospitalized, more severe cases could not be identified during follow-up in their database. Later emerging supporting publications from varying populations, limited to a maximum of 1 year of follow-up, point toward a considerably lower, but still elevated, risk of VTE in patients who were never hospitalized [18,19].

Given the uncertainty of long-term risk of VTE after COVID-19, we identified all recorded infections from national Swedish registers, which were divided into patients initially hospitalized for COVID-19 and followed after discharge and individuals with COVID-19 without hospitalization, and compared them to a non-COVID-19–exposed group randomly selected from the Swedish population and matched for age, sex, and week of COVID-19 diagnosis. The aim of this study was to investigate long-term risk of VTE, separately for PE and DVT after COVID-19, focusing on late VTE risk starting 60 days after patients were indexed in the study.

## 2 Methods

### 2.1 Data sources

By linking multiple nationwide registries, we collected data on hospitalizations, hospital outpatient visits, and deaths from the Swedish National Patient and Cause of Death Registers, kept by the National Board of Health and Welfare (NBHW). The NBHW also registers data on assisted living (including home care and living in a long-term care facility, as previously described in detail) [20]. Information on education, income, and country of origin was collected from Statistics Sweden. Positive SARS-CoV-2 tests using polymerase chain reaction were retrieved from the surveillance system for communicable diseases in Sweden (SmiNet).

### 2.2 Study design and definition of exposed and matched nonexposed populations

**Figure 1:**
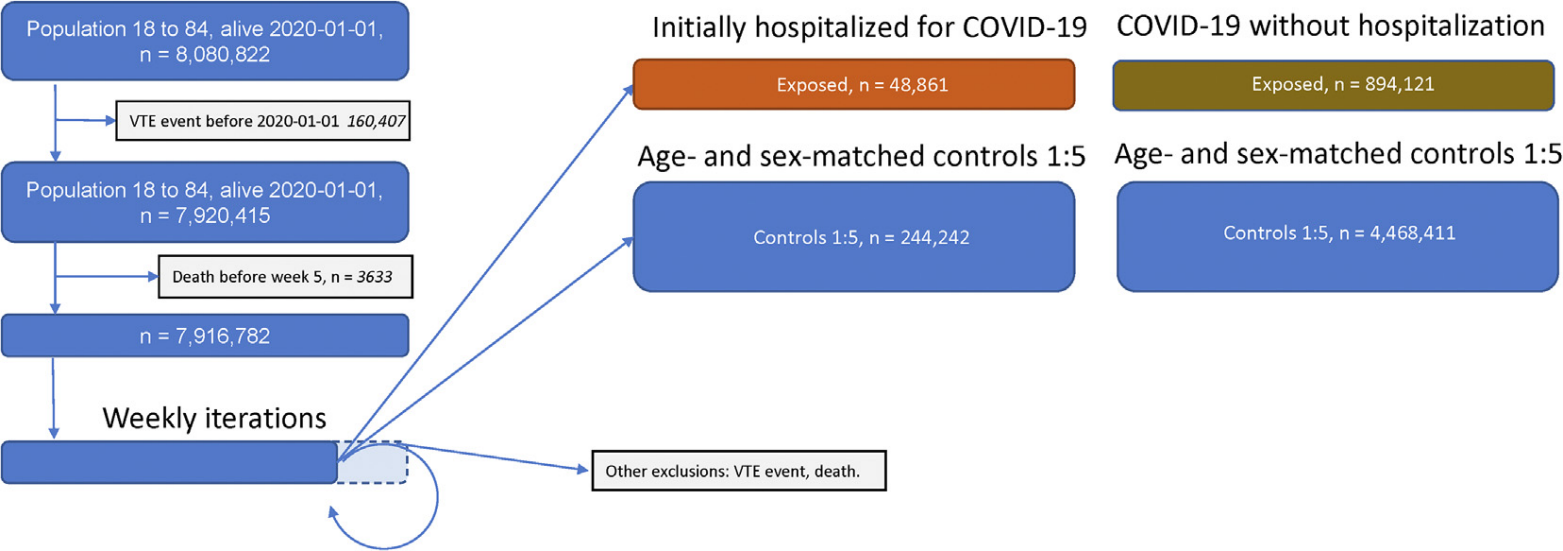
Inclusion, exclusion, and recruitment chart for cases and controls. VTE, venous thromboembolism.

The exposed groups were derived from all Swedish residents aged 18 to 84 years, alive on January 1, 2020, with no prior diagnosis of VTE, and either a laboratory-confirmed diagnosis of SARS-CoV-2 or a hospital discharge code of U071 or U072 according to the International Classification of Diseases. For each COVID-19 case, 5 nonexposed individuals with neither prior VTE diagnosis nor positive polymerase chain reaction test prior to matching were randomly selected from the Total Population Register. Inclusion, exclusion, and recruitment of the cohort are presented in Figure 1. Matching was performed for sex, year of birth, and week of diagnosis of COVID-19 (detailed procedure in Supplementary Figure S1). Weekly matching procedures were performed to correct for potential confounding by seasonal variation in transmission, increasing immunity from infection or vaccination, prevailing regional and national nonpharmaceutical interventions, and availability of testing (which was less systematic in the early months of the pandemic).

Inclusion in the study was between February 1, 2020, and September 11, 2021, with follow-up until November 11, 2021, ie, a minimum of 60 days of observation. Information on baseline diagnostic comorbidities and the outcomes that followed was collected from hospital and inpatient registers, which were registered until December 31, 2019. The detailed International Classification of Diseases codes used are presented in Supplementary Table S1.

COVID-19–exposed patients were divided into those initially hospitalized for COVID-19 and those with COVID-19 without hospitalization. Hospitalizations were considered due to COVID-19 if COVID-19 was the principal diagnosis or COVID-19 was a contributory diagnosis with the principal diagnosis likely to be COVID-19–related (listed in Supplementary Table S2). For multiple hospitalizations in 1 patient, only data from 1 hospitalization were accounted for in the following order: COVID-19 as a principal diagnosis, COVID-19 as a contributory diagnosis with an acceptable principal diagnosis.

For patients initially hospitalized for COVID-19, the index time was defined from the admission date of the first (initial) hospitalization for COVID-19, and for those with COVID-19 without hospitalization, it was defined from the date of laboratory-confirmed SARS-CoV-2 infection. Subjects were followed until the first event of outcome as defined below, death, end of follow-up, or, in the case of nonexposed individuals, a COVID-19 diagnosis.

The Charlson Comorbidity Index (CCI) for register-based research was calculated as described previously [21] with modifications according to data availability (Supplementary Table S3). Data on country of origin were dichotomized into patients born in a Nordic country (Sweden, Denmark, Finland, Iceland, or Norway) or any other country. Information on ethnicity and race is not registered in Swedish population records and, therefore, not available. Information on care for the elderly or disabled was dichotomized into either independent or assisted living. Education was categorized as compulsory (≤9 years), 10 to 12 years, or college/university education.

### 2.3 Outcomes and variables assessed

Outcome measures were diagnosis of PE (I26) or DVT (I801-I809) or VTE defined as either PE or DVT, whichever came first, registered until November 11, 2021, allowing at least 60 days of follow-up of all individuals in the study. The total number of VTE was thus slightly lower than the sum of PE and DVT. Diagnoses included inpatient, outpatient, and cause of death registries. Total mortality was analyzed as a separate event and included all deaths irrespective of prior or concomitant VTE diagnoses.

### 2.4 Statistical analysis

By definition, missing data on specific comorbidities were coded as absence of the relevant comorbidity. Missing data on living conditions were assumed to be independent living. Missing data on the variables born in Nordic countries and education were imputed using multivariate imputation by chained equations [22] before statistical analyses. Variables included in the multivariate imputation by chained equations algorithm were age, sex, education, born in Nordic countries, need for care, and baseline comorbidities. In baseline tables, data are reported without imputation (Table 1; Supplementary Table S4).

**Table 1:** Demographic baseline data for COVID-19–exposed and matched nonexposed individuals.

Variables	Initially hospitalized for COVID-19	Nonexposed^a^	COVID-19 without hospitalization	Nonexposed^a^
	*n* = 48,861	*n* = 244,242	*n* = 894,121	*n* = 4,468,411
Sociodemographic				
Age (y), mean (SD)	60.6 (15.3)	60.6 (15.3)	41.4 (14.9)	41.4 (14.9)
Age (y), group (%)				
18-54	16,340 (33.4)	81,694 (33.4)	711,071 (79.5)	3,553,317 (79.5)
55-64	11,024 (22.6)	55,114 (22.6)	121,894 (13.6)	609,393 (13.6)
65-74	10,517 (21.5)	52,572 (21.5)	42,537 (4.8)	212,647 (4.8)
75-84	10,980 (22.5)	54,862 (22.5)	18,619 (2.1)	93,054 (2.1)
Sex				
Male (%)	29,726 (60.8)	148,587 (60.8)	432,257 (48.3)	2,161,111 (48.4)
Born in Nordic countries (%)^b^				
Yes	31,548 (64.6)	204,993 (83.9)	686,486 (76.8)	3,423,549 (76.6)
Need of care (%)				
Assisted living	4980 (10.2)	7965 (3.3)	10,039 (1.1)	35,087 (0.8)
Professional position (%)				
Blue-collar workers	4910 (10.0)	28,533 (11.7)	124,342 (13.9)	585,952 (13.1)
Essential workers	4562 (9.3)	19,322 (7.9)	121,061 (13.5)	552,168 (12.4)
Hospital staff	3659 (7.5)	15,406 (6.3)	111,386 (12.5)	409,832 (9.2)
School staff	1840 (3.8)	9167 (3.8)	72,958 (8.2)	261,006 (5.8)
Other occupation	9812 (20.1)	67,800 (27.8)	313,740 (35.1)	1,606,720 (36.0)
Retired	12,005 (24.6)	73,030 (29.9)	28,094 (3.1)	172,763 (3.9)
Early retirement	2120 (4.3)	5967 (2.4)	10,468 (1.2)	107,700 (2.4)
Not working	4204 (8.6)	12,286 (5.0)	49,127 (5.5)	376,826 (8.4)
Students	769 (1.6)	4766 (2.0)	52,906 (5.9)	360,357 (8.1)
Education (%)^c^				
≤9 y	12,729 (26.1)	47,595 (19.5)	130,032 (14.5)	686,267 (15.4)
10-12 y	20,699 (42.4)	105,805 (43.3)	392,931 (43.9)	1,842,664 (41.2)
College or university	13,668 (28.0)	84,093 (34.4)	351,548 (39.3)	1,710,215 (38.3)
Baseline comorbidities, *n* (%)				
Diabetes	8364 (17.1)	18,437 (7.5)	23,000 (2.6)	114,425 (2.6)
Hypertension	12,889 (26.4)	39,006 (16.0)	33,018 (3.7)	162,774 (3.6)
Atrial fibrillation	4003 (8.2)	12,025 (4.9)	8924 (1.0)	40,466 (0.9)
Dementia	1204 (2.5)	2194 (0.9)	3522 (0.4)	5919 (0.1)
COPD	3392 (6.9)	6491 (2.7)	6141 (0.7)	32,996 (0.7)
Heart failure	2986 (6.1)	5493 (2.2)	3779 (0.4)	19,101 (0.4)
Cancer	1932 (4.0)	6964 (2.9)	10,378 (1.2)	52,200 (1.2)
Obesity (registered diagnosis)	2360 (4.8)	4776 (2.0)	21,833 (2.4)	99,101 (2.2)
Myocardial infarction	2867 (5.9)	9210 (3.8)	6587 (0.7)	33,423 (0.7)
CCI weighted, mean (SD)	2.00 (2.11)	1.31 (1.73)	0.73 (1.24)	0.72 (1.24)

COPD, chronic obstructive pulmonary disease; CCI, Charlson Comorbidity Index.

a The nonexposed group represent matched (1:5) population–derived subjects for comparison.

b Missing data for born in Nordic countries were 0.4%.

c Missing data for education were 4.6%.

Incident rates were calculated as number of events divided by the total time at risk because matching data were not age standardized. Outcomes were analyzed as VTE and as PE and DVT separately. Furthermore, PE was analyzed among patients without any concurrent DVT in order to reduce the risk of diagnostic bias for PE (Supplementary Table S5). The outcome of VTE was evaluated during initial hospitalization for patients hospitalized for COVID-19, early (0 to <60 days) for all patients, late (60 to <180 days), and long term (from 180 days) until the end of follow-up for all patients. Registrations within 60 days included the sum of VTE during first hospitalization and VTE occurring after discharge from first hospitalization in patients hospitalized for COVID-19 and all VTE events in patients with COVID-19 without hospitalization. Age was categorized into 4 groups: 18 to 54, 55 to 64, 65 to 74, and 75 to 84 years. We restricted the analysis to individuals aged <85 years due to potential underdiagnosis of VTE in elderly, often frail, individuals with multiple medical conditions.

Unadjusted cumulative incidence with competing risk was calculated by the method of Fine and Gray [23]. Events were defined as VTE (including deaths with concomitant VTE diagnosis) or death without VTE. Due to the low rate of VTEs and deaths in the group with COVID-19 without hospitalization, cumulative incidence was calculated for each outcome separately without competing risk. Cox regression models were used to estimate hazard ratios (HRs) and 95% CIs for outcomes comparing the 2 groups of COVID-19 exposed to their matched controls while adjusting for potential confounders. Cox models were applied for patients initially hospitalized for COVID-19 (at 60 to <180 and ≥180 days) and patients with COVID-19 without hospitalization (0 to <60, 60 to <180, and ≥180 days). Cox models could not reliably be applied during the first 60 days in patients initially hospitalized for COVID-19 for several reasons: competing risk with death (as evident by the cumulative incidence plots) as the exact date for a VTE diagnosis was not recorded during hospitalization (only date of admission and discharge were available).

Two models were developed: for model 1, adjustments were made for age and sex. Model 2 was a multivariable-adjusted model for age, sex, obesity, hypertension, need for assisted living, born in Nordic countries, education, and CCI. Most comorbidities (except hypertension, VTE, and obesity) were included in the CCI scale and, therefore, discarded in analyses in which the role of this index was assessed. For completeness, results of model 1 were presented for subjects with COVID-19 who were not hospitalized, as listed in Supplementary Table S6. We have previously created models adjusting for individual comorbidities compared with CCI as a collective covariate and found very similar results, although individual comorbidities were hampered by comparatively low rates, and for that reason, CCI was chosen in this study [21]. All statistical calculations were performed using R software, version 4.0.3 (http://www.R-project.org).

### 2.5 Ethics statement

The study conforms to the principles outlined in the Helsinki Declaration. All data were linked by the NBHW, after which personal identifiers were removed and replaced by a code. The project was approved by the Swedish Ethical Review Authority. Because pseudonymized data were used, consent was not applicable.

## 3 Results

### 3.1 Baseline data

Among 8,080,822 Swedish residents aged 18 to 84 years, COVID-19 was identified in 48,861 patients initially hospitalized for COVID-19 and 894,121 patients with COVID-19 without hospitalization, with no prior VTE (Figure 1; Supplementary Figure S1). The 2 exposed subgroups were matched with nonexposed comparators without a history of VTE or COVID-19, with 244,242 and 4,468,411 individuals selected as matches for follow-up of patients initially hospitalized for COVID-19 and patients with COVID-19 without hospitalization, respectively. The group of patients hospitalized for COVID-19, compared to those without hospitalization, were markedly older, more often men, and born in non-Nordic countries (Table 1). Compared to nonexposed patients, they had more comorbidities, were more often in need of care, were born in non-Nordic countries, and were retired/nonworking, with lower education. For individuals with COVID-19 without hospitalization, the exposed and nonexposed groups were well balanced regarding comorbidities as well as occupational and educational levels but with a higher proportion of hospital and school staff among those exposed to COVID-19.

### 3.2 Incident VTE

#### 3.2.1 Overview

**Figure 2:**
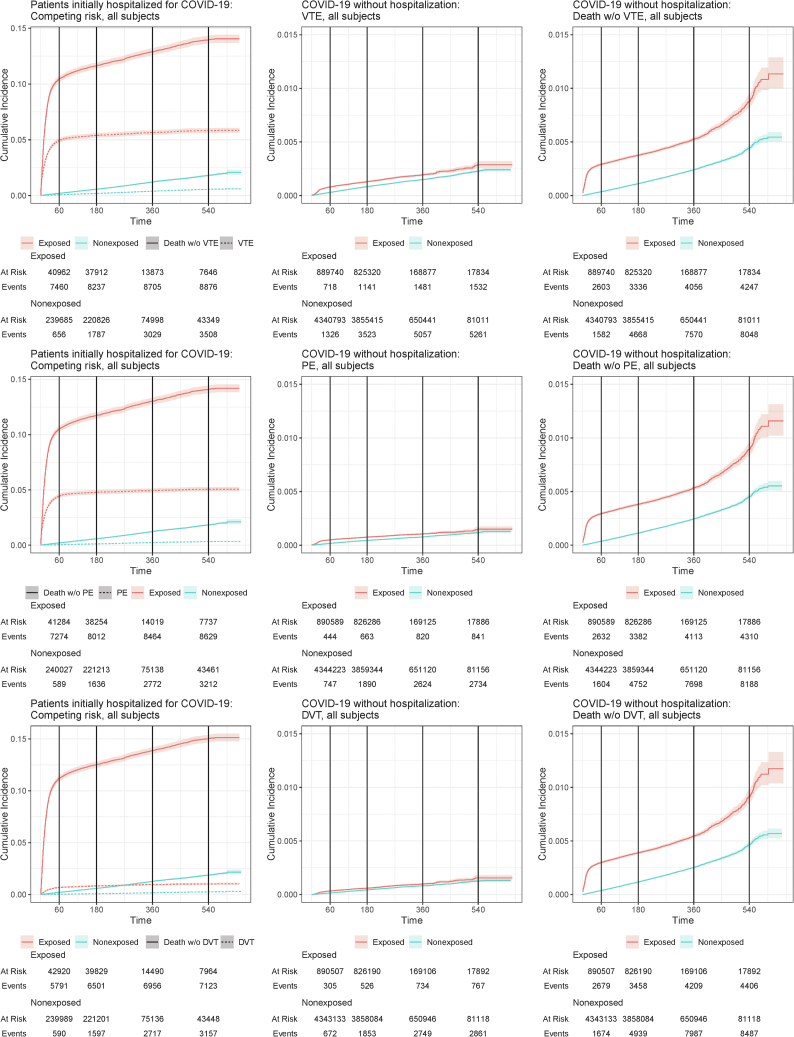
Cumulative incidence curves for venous thromboembolism among patients in COVID-19–exposed and nonexposed groups. DVT, deep venous thrombosis; PE, pulmonary embolism; VTE, venous thromboembolism.

We identified 2380 VTE events in the group of patients initially hospitalized for COVID-19 occurring during follow-up within the first 60 days, the majority of which (1828/2380, 76.8%) were diagnosed with PE during the initial hospital stay (Table 2). Cumulative incidence curves for VTE (Figure 2) show a markedly increased event probability for all outcomes among patients hospitalized for COVID-19, particularly for PE, compared with that in individuals with COVID-19 without hospitalization and the nonexposed groups, with the highest probability early after index time and peaking within 1 to 2 months, followed by a sharp change to a gentler slope after 60 days. Individuals with COVID-19 without hospitalization were much less affected at long-term follow-up, with a VTE event probability closer to that of the nonexposed group. For specific events (VTE, PE, and DVT) per age group, the pattern was similar (Supplementary Figures S2–S7).

**Table 2:** Thromboembolic events in patients hospitalized for COVID-19 (exposed) and matched subjects without COVID-19 (nonexposed) groups.

Age (y)	COVID-19 status	Early (0 to <60 d)				Late (60 to <180 d)					Long term (from 180 d)				
		*N*^a^	During initial hospitalization	After hospitalization^b^	Total	*N*^c^	Events	Event rate (per 1000 y)	HR (CI) Model 1^d^	HR (CI) Model 2^e^	*N*^c^	Events	Event rate (per 1000 y)	HR (CI) Model 1^d^	HR (CI) Model 2^e^
VTE															
18-54	Exposed	16,242	520	111	631	15,396	38	7.8	6.86 (4.21-11.17)	6.58 (3.95-10.98)	13,935	20	2.93	2.72 (1.57-4.72)	2.63 (1.55-4.45)
	Nonexposed	81,620		14	14	79,952	28	1.12			69,867	35	1.09		
55-64	Exposed	10,953	523	80	603	9879	73	23.08	10.65 (7.17-15.83)	10.62 (7.05-16.00)	9279	26	5.66	2.22 (1.40-3.52)	2.24 (1.40-3.59)
	Nonexposed	55,000		20	20	54,023	37	2.15			50,072	62	2.63		
65-74	Exposed	10,381	531	100	631	8350	58	21.81	4.80 (3.38-6.81)	4.08 (2.83-5.89)	7856	25	6.58	1.53 (0.98-2.36)	1.27 (0.80-2.02)
	Nonexposed	52,407		45	45	51,813	73	4.38			49,285	105	4.41		
75-84	Exposed	10,832	406	109	515	7336	51	21.98	3.18 (2.29-4.42)	2.95 (2.09-4.17)	6842	34	9.91	1.65 (1.14-2.38)	1.51 (1.03-2.20)
	Nonexposed	54,636		63	63	53,896	117	6.75			51,601	160	6.02		
Total	Exposed	48,408	1,980	400	2,380	40,961	220	16.91	5.37 (4.48-6.43)	5.05 (4.19-6.1)	37,912	105	5.63	1.85 (1.49-2.30)	1.73 (1.39-2.16)
	Nonexposed	243,663		142	142	239,684	255	3.35			220,825	362	3.41		
PE															
18-54	Exposed	16,264	477	74	551	15,497	19	3.87	9.51 (4.43-20.41)	8.11 (3.53-18.64)	14,045	10	1.45	3.37 (1.50-7.59)	2.85 (1.28-6.36)
	Nonexposed	81,665		5	5	80,005	10	0.4			69,930	14	0.43		
55-64	Exposed	10,976	482	65	547	9953	63	19.77	18.70 (11.07-31.58)	19.18 (11.21-32.84)	9358	20	4.31	2.95 (1.71-5.11)	3.09 (1.75-5.45)
	Nonexposed	55,058		7	7	54,092	18	1.04			50,150	36	1.52		
65-74	Exposed	10,423	500	87	587	8420	46	17.14	5.44 (3.61-8.18)	4.64 (3.02-7.15)	7932	16	4.17	1.63 (0.94-2.83)	1.36 (0.75-2.47)
	Nonexposed	52,486		27	27	51,904	50	3			49,384	62	2.6		
75-84	Exposed	10,881	369	92	461	7413	37	15.77	3.37 (2.27-5.00)	3.08 (2.02-4.68)	6919	22	6.34	1.93 (1.22-3.08)	1.74 (1.07-2.83)
	Nonexposed	54,736		33	33	54,025	79	4.54			51,748	88	3.3		
Total	Exposed	48,544	1,828	318	2,146	41,283	165	12.57	6.5 (5.22-8.09)	6.05 (4.80-7.62)	38,254	68	3.61	2.18 (1.66-2.87)	2.01 (1.51-2.68)
	Nonexposed	243,945		72	72	240,026	157	2.06			221,212	200	1.88		
DVT															
18-54	Exposed	16,312	58	45	103	15,966	25	4.95	6.39 (3.52-11.61)	6.70 (3.63-12.38)	14,452	11	1.56	2.41 (1.16-4.99)	2.56 (1.29-5.08)
	Nonexposed	81,645		10	10	79,980	19	0.76			69,896	21	0.65		
55-64	Exposed	10,998	58	26	84	10,393	21	6.29	4.97 (2.73-9.03)	4.58 (2.47-8.48)	9810	8	1.65	1.31 (0.60-2.87)	1.21 (0.55-2.70)
	Nonexposed	55,050		14	14	54,075	22	1.28			50,127	30	1.27		
65-74	Exposed	10,468	52	23	75	8855	14	4.95	3.06 (1.61-5.81)	2.62 (1.35-5.09)	8,361	11	2.73	1.35 (0.70-2.59)	1.13 (0.58-2.21)
	Nonexposed	52,483		19	19	51,911	27	1.62			49,412	50	2.09		
75-84	Exposed	10,925	46	24	70	7706	17	6.96	2.71 (1.55-4.75)	2.70 (1.54-4.74)	7206	16	4.44	1.44 (0.84-2.45)	1.41 (0.82-2.43)
	Nonexposed	54,751		34	34	54,022	44	2.53			51,765	82	3.07		
Total	Exposed	48,703	214	118	332	42,920	77	5.64	4.08 (3.06-5.44)	3.97 (2.96-5.33)	39,829	46	2.36	1.50 (1.08-2.07)	1.46 (1.05-2.01)
	Nonexposed	243,929		77	77	239,988	112	1.47			221,200	183	1.72		

DVT, deep venous thrombosis; HR, hazard ratio; PE, pulmonary embolism; VTE, venous thromboembolism.

a Excluding patients with an event occurring between January 1, 2020, and the diagnosis of COVID-19.

b Refers to events occurring after discharge from first hospitalization. The nonexposed groups include all events occurring during the full 60-day period.

c Numbers that were alive, had not experienced an event, and were still in follow-up.

d Model 1 adjusted for age and sex.

e Model 2 adjusted for age, sex, obesity, hypertension, need for assisted living, born in Nordic country, education, and Charlson Comorbidity Index.

### 3.3 Late and long-term follow-up for patients initially hospitalized for COVID-19

During the late follow-up period (60 to <180 days), the HRs for VTE for patients initially hospitalized for COVID-19, compared with those of nonexposed patients, were generally higher among younger than among older individuals, most notably for PE (Table 2). For example, after full adjustment (for age, sex, obesity, hypertension, need for assisted living, born in Nordic country, education, and CCI) the HR for PE among individuals aged 55 to 64 years was 19.18 (CI, 11.21-32.84), with the corresponding HR in those aged 75 to 84 years being 3.08 (CI, 2.02–4.68) and that collectively across all age groups being 6.05 (CI, 4.80-7.62) (Table 2). The corresponding HR for DVT across all age groups was 3.97 (CI, 2.96-5.33). During long-term follow-up (from 180 days) the incident VTE hazard for the exposed group hospitalized for COVID-19 vs that for the nonexposed group was less pronounced, with a total HR for PE of 2.01 (CI, 1.51-2.68) and lower HR for DVT and VTE. The hazard for PE after 180 days tended to be higher among younger than among older patients and highest at ages 55 to 64 years (HR, 3.09; CI, 1.75-5.45).

### 3.4 Late and long-term follow-up for subjects with COVID-19 without hospitalization

Among the 894,121 individuals with COVID-19 without hospitalization, the rates of VTE, PE, and DVT were considerably lower than those for the patients initially hospitalized for COVID-19 (Tables 2 and 3; Supplementary Table S6). Overall, from 60 days after index, follow-up of the group with COVID-19 without hospitalization did not display any independently elevated hazard of DVT or PE (Table 3). There was a slightly increased HR for PE in the late phase (60 to <180 days) but only among those aged 65 to 74 years (HR, 1.70; CI, 1.22-2.36) and from 180 days solely for VTE at age 18 to 54 years (HR, 1.20; CI, 1.02-1.40).

**Table 3:** Thromboembolic events in subjects with COVID-19 without hospitalization (exposed) and without COVID-19 (nonexposed) groups.

Age (y)	COVID-19 status	Early (0 to <60 d)			Late (60 to <180 d)				Long term (from 180 d)			
		*N*^a^	Total events	HR (CI) Model 2^b^	*N*^c^	Events	Event rate (per 1000 y)	HR (CI) Model 2^b^	*N*^c^	Events	Event rate (per 1000 y)	HR (CI) Model 2^d^
VTE												
18-54	Exposed	710,570	319	3.07 (2.66-3.54)	710,126	206	0.92	1.10 (0.95-1.28)	653,695	198	0.9	1.20 (1.02-1.40)
	Nonexposed	3,550,407	502		3,446,525	891	0.84		3,022,616	735	0.76	
54-64	Exposed	121,640	183	3.44 (2.83-4.17)	121,298	91	2.32	0.86 (0.68-1.08)	116,428	93	2.24	1.01 (0.81-1.27)
	Nonexposed	608,108	260		593,679	500	2.65		550,608	431	2.3	
65-74	Exposed	42,360	126	3.76 (2.96-4.77)	41,715	75	5.6	1.47 (1.14-1.90)	39,740	59	4.24	1.10 (0.83-1.47)
	Nonexposed	211,901	159		209,148	257	3.86		196,413	255	3.77	
75-84	Exposed	18,472	90	3.86 (2.88-5.17)	16,600	57	10.88	1.27 (0.94-1.70)	15,457	43	6.73	1.05 (0.75-1.47)
	Nonexposed	92,609	112		91,367	234	8.09		85,665	216	5.74	
Total	Exposed	893,042	718	3.32 (3.01-3.66)	889,739	429	1.52	1.10 (0.99-1.22)	825,320	393	1.39	1.11 (0.99-1.24)
	Nonexposed	4,463,025	1033		4,340,719	1882	1.4		3,855,302	1637	1.3	
PE												
18-54	Exposed	710,889	192	4.04 (3.32-4.91)	710,568	83	0.37	1.11 (0.88-1.41)	654,205	76	0.34	1.16 (0.90-1.50)
	Nonexposed	3,552,215	227		3,448,541	359	0.34		3,024,882	289	0.3	
54-64	Exposed	121,777	106	3.56 (2.75-4.61)	121,503	52	1.32	0.97 (0.72-1.31)	116,657	44	1.06	0.98 (0.70-1.35)
	Nonexposed	608,788	143		594,449	262	1.39		551,527	212	1.13	
65-74	Exposed	42,445	82	3.83 (2.83-5.17)	41,834	47	3.5	1.70 (1.22-2.36)	39,874	28	2.01	0.95 (0.63-1.44)
	Nonexposed	212,270	97		209,565	139	2.08		196,887	140	2.07	
75-84	Exposed	18,541	64	4.32 (3.02-6.19)	16,683	37	7.02	1.29 (0.90-1.86)	15,550	31	4.82	1.36 (0.91-2.04)
	Nonexposed	92,804	70		91,594	150	5.17		85,936	123	3.26	
Total	Exposed	893,652	444	3.85 (3.38-4.38)	890,588	219	0.77	1.17 (1.01-1.35)	826,286	179	0.63	1.08 (0.92-1.28)
	Nonexposed	4,466,077	537		4,344,149	910	0.67		3,859,232	764	0.61	
DVT												
18-54	Exposed	710,740	141	2.22 (1.82-2.72)	710,465	130	0.58	1.06 (0.87-1.28)	654,067	134	0.61	1.21 (1.00-1.47)
	Nonexposed	3,551,362	310		3,447,596	582	0.55		3,023,786	490	0.51	
54-64	Exposed	121,744	83	2.98 (2.26-3.92)	121,494	44	1.12	0.71 (0.51-0.99)	116,657	58	1.4	1.06 (0.80-1.41)
	Nonexposed	608,648	139		594,297	278	1.47		551,337	256	1.37	
65-74	Exposed	42,444	50	3.42 (2.37-4.91)	41,851	30	2.23	1.12 (0.75-1.66)	39,900	35	2.5	1.26 (0.87-1.84)
	Nonexposed	212,233	73		209,538	135	2.02		196,855	131	1.93	
75-84	Exposed	18,545	31	3.23 (2.02-5.18)	16,696	23	4.36	1.31 (0.82-2.08)	15,566	15	2.33	0.74 (0.43-1.27)
	Nonexposed	92,835	47		91,629	91	3.13		85,993	106	2.81	
Total	Exposed	893,473	305	2.62 (2.28-3.02)	890,506	227	0.8	0.99 (0.86-1.15)	826,190	242	0.86	1.14 (0.99-1.31)
	Nonexposed	4,465,078	569		4,343,060	1,086	0.8		3,857,971	983	0.78	

DVT, deep venous thrombosis; HR, hazard ratio; PE, pulmonary embolism; VTE, venous thromboembolism.

a Excluding patients with events occurring between January 1, 2020, and day of diagnosis of COVID-19.

b Model 1 with adjustment limited to age and sex is presented in Supplementary Table S6 for completeness.

c Numbers that were alive, had not experienced an event, and was still in follow-up.

d Model 2 is adjusted for age, sex, obesity, hypertension, need for assisted living, born in a Nordic country, education, and Charlson Comorbidity Index.

### 3.5 Consideration of bias and sensitivity analyses

To address the additional impact of comorbidities in patients hospitalized for COVID-19, we analyzed patients with a CCI of 0, ie, no comorbidities. We identified 19,624 individuals among those initially hospitalized for COVID-19 and 624,933 among those not hospitalized with a CCI of 0, corresponding to 62,263 and 2,242,124 from the matched nonexposed controls, respectively. For patients hospitalized for COVID-19 with a CCI of 0, the event rates (per 1000 years) for late follow-up (60 to <180 days) of VTE were slightly lower than those for the entire group (15.05 vs 16.91, respectively), with an even more marked difference between the corresponding nonexposed groups (1.35 vs 3.35, respectively), resulting in HRs (CCI = 0 vs all patients) of 9.70 (CI, 6.19-15.22) vs 5.37 (CI, 4.48-6.43), respectively, for model 1 (Table 2; Supplementary Table S7). The corresponding event rates for long-term follow-up (from 180 days) were 3.46 vs 5.63, respectively, in the exposed and 1.51 vs 3.41, respectively, in the nonexposed group with a CCI of 0, with consequent HRs of 2.16 (CI,1.30-3.57) vs 1.85 (CI,1.49-2.30), respectively (Table 2; Supplementary Table S7). For subjects with COVID-19 without hospitalization and a CCI of 0, the event rates were overall lower than those in the complete group, accompanied by similarly lower event rates in the nonexposed group with a CCI of 0, resulting in HRs similar to those in the entire cohort (Table 3; Supplementary Table S8).

To elucidate the influence of competing risk by death, we present mortality data for the respective time periods, showing very high numbers for patients initially hospitalized with COVID-19, ie, during the early period studied (0 to <60 days), with an HR of 63.5 (CI, 57.93-69.61) compared with 8.28 (CI, 7.78-8.82) for subjects with COVID-19 who were not hospitalized. The following time period showed considerably lower numbers: 60 to <180 days, with an HR of 4.73 (CI, 4.28-5.23) for the initially hospitalized, falling successively over time (>180 days), with an HR of 2.79 (CI, 2.54-3.07). The nonhospitalized cohort had lower mortality. The number of events from the same original population of initially hospitalized patients (*n* = 48,861) for the different time periods were 5518, 661, and 608, respectively (Supplementary Table S9).

The hazard of PE without concomitant DVT showed an HR pattern similar to that reported for PE overall: at out-of-hospital follow-up of patients hospitalized for COVID-19 in the late period (60 to <180 days) at age 55 to 64 with HR 18.84 (CI, 10.70-33.19) and for the total cohort HR 6.25 (CI, 4.88-8.01) (Supplementary Table S5).

## 4 Discussion

In this Swedish population-based cohort study, all patients with confirmed COVID-19 infection were included in long-term outpatient follow-up and analyzed as 2 groups: patients initially hospitalized for COVID-19 and individuals with COVID-19 without hospitalization. Comparisons were made with matched individuals without known COVID-19 exposure. During the early phase, there was a dramatic increase in VTE, dominated by PE, relative to that in nonexposed individuals, which tapered rapidly (<60 days). The incidence of VTE was highest among patients hospitalized for COVID-19 [24]. Importantly, among individuals with COVID-19 without hospitalization the risk of VTE had already reverted to normal at 60 days of follow-up after COVID-19 and was subsequently comparable with that in the background population after adjustment. Patients who had been hospitalized for COVID-19, however, still carried a heightened hazard of VTE after the first 60 days, particularly with respect to PE and in the younger subgroups of the population but at a much lower level than in the first period. This is the first study providing long-term, nationwide follow-up of long-term VTE outcomes in both patients hospitalized for COVID-19 and nonhospitalized individuals of an entire country compared with that in a matched population–based nonexposed group.

### 4.1 General

After the early phase (≥60 days), the proportions between incident PE and DVT became more evenly distributed, but a larger excess hazard of PE relative to that to DVT remained, particularly for patients who were initially hospitalized for COVID-19 during the 60 to <180 days of the follow-up period compared with that in the nonexposed patients. The proportional rise in PE over VTE has not been a consistent observation [1,2,4,5,11,12], but in this large observational study, including long-term follow-up, we were able to confirm a strong preponderance of PE in relation to DVT. Mechanistic explanations may involve inflammation of the pulmonary vasculature, endothelial disruption, and activation of coagulation [[25], [26], [27]]. Detection bias due to severe respiratory symptoms has been proposed but might equally lead to underdiagnosis as pulmonary involvement may be attributed to the natural course of COVID-19 [[28], [29], [30]]. In our study, a similar pattern for PE and PE without DVT supports the absence of detection bias for PE. Importantly, the dramatic increase in VTE during hospitalization and protective effects of anticoagulation (AC) [1,31,32] led to prophylactic implementation of in-hospital AC treatment early during the pandemic [1].

### 4.2 Patients initially hospitalized for COVID-19 vs COVID-19 without hospitalization

Consistent with prior studies, we observed that cardiometabolic comorbidities, older age, and male sex were associated with a more severe initial course of COVID-19, increased risk of hospitalization [[33], [34], [35], [36], [37]], and dramatic increase in the hazard of VTE [38]. Likely, the propensities for VTE and more severe COVID-19 are determined by host factors such as traditional CVD risk factors [15,39]. The rapid reduction in the HR of VTE over time during follow-up from 60 to <180 days and >180 days can also be observed in the cumulative incidence curves as a lower event probability of VTE in subjects with COVID-19 without hospitalization and more rapid restoration to normal. The predominance of PE over DVT, particularly in patients initially hospitalized for COVID-19, may reflect the lungs as the major locus of inflammation and hypercoagulation [26,27,40]. The affected individuals with COVID-19 without hospitalization and the matched, nonexposed patients were demographically similar, except for a higher proportion of hospital and school staff among infected individuals, consistent with prior findings [[41], [42], [43]].

### 4.3 Course at late (60 to <180 days) and long-term (≥180 days) follow-up

A high incidence of intravascular clotting and intense inflammation has been repeatedly reported during acute COVID-19 and has raised concern for long-term pulmonary vascular function and threat of complications relative to VTE [15]. However, we found that the general risk of VTE from day 60 and onward in subjects with COVID-19 without hospitalization was comparable to that in the background population, albeit with some variation in risk between age groups, but without any consistent pattern. By contrast, follow-up after discharge of the patients initially hospitalized for COVID-19 held an extended and markedly heightened hazard for VTE over a lengthy follow-up after adjustment for several comorbidities. The rapidly reduced incidence in the group with COVID-19 without hospitalization may have resulted from the decrease of inflammatory activity after the acute phase of COVID-19 as inflammation with activation of hematologic and immunologic pathways has been linked to VTE [[25], [26], [27]]. A fast decrease in risk of VTE after COVID-19 was also observed in a large cohort of patients registered in primary care general practice covering England and Wales [18]. However, even after extensive adjustment, this study still showed a considerably elevated hazard of VTE for nonhospitalized patients 8 weeks after diagnosis. One may speculate that patients already registered in primary practice may exhibit prior health problems predisposing them to thromboembolism, while we compared with controls from the total population. Altogether, our findings offer reassurance in suggesting a long-term normalized risk of VTE after the first 60 days in individuals with COVID-19 without hospitalization.

### 4.4 Consideration of bias

The risk of bias due to comorbidities, frailty, and vulnerability being more common in patients hospitalized for COVID-19 and not fully captured by statistical modeling was considered. We performed a sensitivity analysis including only patients with a CCI of 0, ie, without comorbidities, and observed a slightly lower event rate in patients initially hospitalized for COVID-19 with a CCI of 0 compared with that in the complete hospitalized cohort. The minor difference in event rates indicates that comorbidities were of comparatively minor importance for incident VTE, leaving the severity of COVID-19 infection as the likely cause. Notably, due to an even lower event rate in nonexposed patients with a CCI of 0 than in the complete cohort, the resulting HR increased for hospitalized patients with a CCI of 0 compared with that in the complete cohort, also indicating the severity of infection as the main explaining factor. Also, for individuals with COVID-19 without hospitalization, event rates were lower for those with a CCI of 0 than for the entire population but similar across groups producing stable HRs, similarly indicating that comorbidities were not the major explanatory factor for VTE outcomes.

To address the potential impact of unbalanced mortality between the comparator groups on VTE outcomes (as hospitalization is an independent predictor of mortality and the comparator groups were not matched specifically to this background), we examined the relationships between mortality in the different groups and over time. The highest mortality within 60 days occurred in the group initially hospitalized for COVID-19 (*n* = 5518), plummeted during 60 to <180 days, and was even lower in nonhospitalized patients and over a long time. Thus, the substantial drop in mortality, with resulting few events after day 60, does not indicate any considerable effect of competing risk of death or loss of person-time at risk on any long-term VTE outcome.

### 4.5 Strengths and limitations

The strength of our data is the comprehensive national coverage and timeline of all patients with a confirmed diagnosis of COVID-19 complicated by VTE in Sweden. Complete follow-up from the acute phase to the long-term phase, covering all patients initially hospitalized for COVID-19 and subjects with COVID-19 without hospitalization, strengthens the generalizability of the results regarding the late risks of VTE in a population of similar composition. The sequential generation of a time-matched, nonexposed population is likely to minimize confounding external factors regarding the course of the pandemic. Also, our study required a laboratory-confirmed diagnosis of COVID-19, thereby ensuring the reliability of the diagnosis.

However, our study also has some weaknesses as several sampling biases exist regarding higher risk in patients hospitalized for COVID-19 relative to the nonexposed patients and hospitalization was not a matching variable: hospitalization *per se* is an independent risk factor for VTE (also after hospital discharge) and is likely to interact with COVID-19, for example, as physicians are more likely to hospitalize patients with comorbidities. Hospitalized patients are more likely to die, leading to only survivors included in the long-term follow-up, and they also carry more comorbidities relative to the nonexposed patients. As nonexposed controls to patients hospitalized for COVID-19 were not themselves hospitalized, it is not possible to separate the risk associated with severe COVID-19 infection from the elevated risk of VTE associated with hospitalization *per se*. This may create a comparison unfairly biased toward a higher risk of VTE in the exposed group, which includes patients with a strong independent risk factor for VTE (other than COVID-19) that is not balanced in the control group. Furthermore, even though CCI was adjusted for, it may not be enough to capture the severity of underlying multimorbidity and frailty and, thus, display an exaggerated HR. We addressed this in a sensitivity analysis, demonstrating only a marginally reduced VTE event rate in hospitalized patients (60 to <180 days) with a CCI of 0, indicating that comorbidities were not likely to be the driver of incident VTE.

As only hospital survivors were included in the long-term follow-up, bias may have affected outcome in both directions. On the one hand, diluting the difference between exposed and nonexposed since patients who developed fatal VTE during hospitalization for COVID-19 were not available for further follow-up. On the other hand, there was a possible elevation of incident VTE in the small fraction of nonexposed patients hospitalized for non-COVID-19–related disease due to lack of prophylactic AC. Additionally, if the elevated all-cause mortality rate in the hospitalized population continued over time, this will contribute to overestimation of long-term HR due to competing risk of death and loss of person-time at risk as death occurs disproportionally more frequently in patients hospitalized for COVID-19. We cannot fully disentangle these considerations. However, mortality fell rapidly and substantially after the first 60 days, indicating no major impact from competing risk of death or loss of person-time at risk on the outcome variables.

Finally, to account for other hospital-related risk factors for either death or VTE, we considered generating a comparison group hospitalized for other medical conditions. However, despite the potential advantages of creating a control group of patients similar to the group with COVID-19, it would, for several reasons, not be feasible to identify an appropriate comparable hospitalized cohort given the marked heterogeneity of the in-hospital patient population. Importantly, during the ongoing pandemic, considerable displacement of less prioritized patient groups prevailed in emergency medical services, which would have introduced additional bias. After careful consideration, we accepted the limitations in the present study as the most rigorous method available.

As a mild limitation, the cohort of patients hospitalized for COVID-19 may have included some patients acquiring COVID-19 while in hospital for other causes, where the infection alone would not have warranted hospitalization. However, only patients with a principal diagnosis of COVID-19 were included in the case definition (Supplementary Table S2). Diagnostic testing was severely restricted during the first months of the pandemic, and early infections were most certainly missed, foremost in the group with COVID-19 without hospitalization. Moreover, there was little clinical detail on the course of disease prior to the diagnosis of VTE or the level of care needed in each case. The vaccination status of the cohort, which may have affected incidence, symptoms, and diagnostics of COVID-19, was unknown. Furthermore, there were no data available on the possible practice of routine screening for DVT, which may have affected detection [4]. Finally, only prior in- and out-patient hospital diagnoses were captured, which will have underestimated comorbidities diagnosed in primary care (such as diabetes, hypertension, and obesity), and there was no information on body weight or lifestyle.

## 5 Conclusions

Our study confirms an extremely elevated risk of VTE, predominantly PE, in the early phase of severe COVID-19, with rapid tapering within 60 days. In contrast to nonpandemic conditions, PE was the predominant VTE. From 60 days and onward, the overall risk of VTE became similar to the background population rate for the group with COVID-19 without hospitalization but remained elevated during long-term out-patient follow-up for the cohort initially hospitalized for COVID-19, particularly for PE, and in the younger age groups. However, there was also substantial attenuation of long-term risk in patients hospitalized for COVID-19. These data indicate, at most, a transient increase in future risk of VTE in patients with COVID-19 without hospitalization, ie, most affected individuals.
