# Supplementary material for: Pulmonary embolism and deep venous thrombosis after COVID-19: long-term risk in a population-based cohort study
Source: Res Pract Thromb Haemost. 2023 Jun 21;7(5):100284. doi: 10.1016/j.rpth.2023.100284 (PMC10284449; doi:10.1016/j.rpth.2023.100284)
Supplement: Supplementary Material [file mmc1.docx]

**Supporting information**

**Long-term risk of pulmonary embolism and deep venous thrombosis after covid-19. A nationwide case-control study.**

Helen Sjöland, Martin Lindgren, Triantafyllia Toska, Per-Olof Hansson, Katarina Glise Sandblad, Christian Alex, Lena Björck, Ottmar Cronie, Jonas Björk, Christina E Lundberg, Martin Adiels, Annika Rosengren

**Table S1. Definitions of baseline comorbidities and diagnostic codes**

| **Diagnosis** | **ICD-9** | **ICD-10** |
| --- | --- | --- |
| Atrial fibrillation | 427D | I48 |
| COPD | 491 | J44 |
| Diabetes | 250 | E10–E14 |
| Dementia | 290 | F00–F09 |
| Heart failure | 428 | I50 |
| Hypertension | 401–405 | I10–I15 |
| Malignancy | 140–208 | C00–C97 |
| Myocardial infarction | 410 | I21 |
| Obesity | 278A, 278B | E65, E66 |
| Stroke (ischemic and haemorrhagic) | 431, 433, 434, 436 | I61–I64 |
| Venous thromboembolism | 415B, 416W, 451 (not 451A) | I26, I80.1-I80.9 |

**Table S2. Principal diagnoses used together with a secondary COVID-19 diagnosis**

| **Included categories** | **ICD-codes for hospitalization** | **ICD-codes for death** |
| --- | --- | --- |
| **COVID-related symptoms** |  |  |
| Cough | R05 |  |
| Abnormalities of breathing | R06 |  |
| Pain in throat and chest | R07 |  |
| Other symptoms and signs involving the circulatory and respiratory system | R09 |  |
| Dizziness and giddiness | R42 |  |
| Fever of other or unknown origin | R50 |  |
| Headache | R51 |  |
| Malaise and fatigue | R53 |  |
| Syncope and collapse | R55 |  |
| **Upper and lower respiratory tract infections** |  |  |
| Acute nasopharyngitis | J00 |  |
| Acute tonsillitis | J03 |  |
| Acute upper respiratory infections of multiple and unspecified sites | J06 |  |
| Influenza due to other identified influenza virus | J10 |  |
| Other viral pneumonia | J128 |  |
| Viral pneumonia, unspecified | J129 |  |
| Pneumonia due to Streptococcus pneumoniae | J13 |  |
| Bacterial pneumonia, not elsewhere classified | J15 |  |
| Pneumonia due to other specified infectious organisms | J168 |  |
| Pneumonia in diseases classified elsewhere | J17 |  |
| Pneumonia, unspecified organism | J18 |  |
| Unspecified acute lower respiratory infection | J22 |  |
| Coronavirus infection, unspecified | B342 |  |
| Other viral infections of unspecified site | B348 |  |
| Viral infection, unspecified | B349 |  |
| Coronavirus as the cause of diseases classified elsewhere | B972 |  |
| Other and unspecified infectious diseases | B99 | B99 |
| **Respiratory disorders** |  |  |
| Pulmonary embolism | I26 |  |
| Acute respiratory distress syndrome | J80 |  |
| Pulmonary edema | J81 |  |
| Pleural effusion not elsewhere classified | J90 |  |
| Respiratory failure, not elsewhere classified | J96 | J96 |
| Respiratory disorders in diseases classified elsewhere | J99 |  |
| **Obstructive Airway Diseases** |  |  |
| Acute bronchitis due to other specified organisms | J208 |  |
| Acute bronchitis, unspecified | J209 |  |
| Acute bronchiolitis due to other specified organisms | J218 |  |
| Acute bronchiolitis, unspecified | J219 |  |
| Other chronic obstructive pulmonary disease | J44 | J44 |
| Asthma | J45 | J45 |
| **Cardiac diseases** |  |  |
| Viral carditis | B332 |  |
| Chronic ischemic heart disease | I25 | I25 |
| Acute pericarditis | I30 | I30 |
| Pericarditis in diseases classified elsewhere | I32 |  |
| Acute myocarditis, unspecified | I40 |  |
| Myocarditis in diseases classified elsewhere | I41 |  |
| Atrial fibrillation and flutter | I48 | I48 |
| Heart failure | I50 | I50 |
| Abnormalities of heartbeat | R00 |  |
| **Kidney disorders** |  |  |
| Acute kidney failure | N17 | N17 |
| Chronic kidney disease | N18 | N18 |
| Unspecified kidney failure | N19 | N19 |
| **Electrolyte disorders** |  |  |
| Other disorders of fluid, electrolyte and acid-base balance | E87 | E87 |

**Table S3. Modifications of Charlson Comorbidity Index**

|  | **ICD9** | | | **ICD10** | | |
| --- | --- | --- | --- | --- | --- | --- |
|  | **Modified** | **Original** | **Difference** | **Modified** | **Original** | **Difference** |
| **Myocardial infarction** | 410, 412 | 410, 412 |  | I21, I22, I252 | I21, I22, I252 |  |
| **Congestive heart failure** | 402A/B/X, 404A/B/X, 425E/F/H/W/X, 428 | 402A/B/X, 404A/B/X, 425E/F/H/W/X, 428 |  | I110, I130/2, I255, I420/6/7/9, I43, I50 | I110, I130/2, I255, I420/6/7/9, I43, I50 |  |
| **Peripheral vascular disease** | 440, 441, 443B/X, 447B | 440, 441, 443B/X, 447B, **557** | Excluded: 557 (Vascular insufficiency of intestine) | I70, I71, I731/8/9, I771, I790/2 | I70, I71, I731/8/9, I771, I790/2, **K55** | Excluded: K55 (Vascular disorders of intestine) |
| **Cerebrovascular disease** | 430-438 | 430-438 |  | G45, I60, I61, I62, I63, I64, I67, I69 | G45, I60, I61, I62, I63, I64, I67, I69 |  |
| **Chronic obstructive pulmonary disease** | 491, 492, 496 | 491, 492, 496 |  | J43, J44 | J43, J44 |  |
| **Chronic other pulmonary disease** | 490, 493-495, 500-508, 516, 517 | 490, 493-495, 500-508, 516, 517 |  | J41, J42, J45-J47, J60-J70 | J41, J42, J45-J47, J60-J70 |  |
| **Rheumatic disease** | 446 | 446, **696A, 710A/B/C/D/E, 714, 719D, 720, 725** | Excluded: 969A (Psoriasis joint), 710A/B/C/D/E (Diffuse diseases of connective tissue), 714 (Rheumatoid arthritis and other inflammatory polyarthropathies), 719D (Palindromic rheumatism), 720 (Ankylosing spondylitis and other inflammatory spondylopathies), 725 (Polymyalgia rheumatica) | M05, M06, M13, M30, M313/4/5/6, M32-M34, M350/1/3, M45 | M05, M06, **M070/1/2/3, M08, M123**, M13, M30, M313/4/5/6, M32-M34, M350/1/3, M45, **M46** | Excluded: M07X (Psoriatic and enteropathic arthropathies), M08 (Juvenile arthritis), M46 ( Other inflammatory spondylopathies), M123 (Palindromic rheumatism) |
| **Dementia** | 290, 294B | 290, 294B, **331A/B/C/X** | Excluded: 331/A/B/C/X (Other cerebral degenerations: Alzheimer's disease, Frontotemporal dementia, Senile degeneration of brain, Cerebral degeneration, unspecified) | F00-F03, F051, **G30-G32** | F00-F03, F051, **G30, G311, GG319** | Included: G310 (Circumscribed brain atrophy), G312 (Degeneration of nervous system due to alcohol), G318 ( Other specified degenerative diseases of nervous system), G32 (Other degenerative disorders of nervous system in diseases classified elsewhere) |
| **Hemiplegia** | 343, **344** | **342**, 343, **344A/B/C/D/E/F** | Excluded: 342 (Hemiplegia and hemiparesis). Included: 344G (Cauda equina syndrome), 344W (Other specified paralytic syndromes), 344X (Paralysis, unspecified) | G80-G83 | **G114,** G80-G82, **G830/1/2/3/8** | Excluded: G114 (Hereditary spastic paraplegia). Included G834 (Cauda equina syndrome), G839 (Paralytic syndrome, unspecified) |
| **Diabetes without chronic complication** | 250A/B/C | 250A/B/C |  | E100/1, E110/1, E120/1, E130/E131, E140/1 | E100/1, E110/1, E120/1, E130/E131, E140/1 |  |
| **Diabetes with chronic complication** | 250D/E/F/G | 250D/E/F/G |  | E102/3/4/5/7, E112-E117, E122-E127, E132-E137, E142-E147 | E102/3/4/5/7, E112-E117, E122-E127, E132-E137, E142-E147 |  |
| **Renal disease** | 403/A/B/X, 583, 585, 586, 588A, V56 | 403/A/B/X, 583, 585, 586, 588A, **V42A, V45B,** V56 | Excluded: V42A (Kidney replaced by transplant), V45B (Postsurgical renal dialysis status) | I120, I131, N18, N19, Z940 | I120, I131, N032-N037, **N052-N057, N11**, N18, N19, **N250,** **Q611-Q614, Z49**, QZ940 | Excluded: N052-N057 (Unspecified nephritic syndrome: Diffuse membranous glomerulonephritis, Diffuse mesangial proliferative glomerulonephritis, Diffuse endocapillary proliferative glomerulonephritis, Diffuse mesangiocapillary glomerulonephritis, Dense deposit disease, Diffuse crescentic glomerulonephritis), N11 (Chronic tubulo-interstitial nephritis), N250 (Renal osteodystrophy), Q611-Q614 (Polycystic kidney: autosomal recessive, autosomal dominant, unspecified. Renal dysplasia), Z49 (Dialysis) |
| **Mild liver disease** | ***NA*** | **070, 571C/E/F, 573** | Excluded: 070 (Viral hepatitis), (Chronic liver disease and cirrhosis: Alcoholic cirrhosis, Chronic hepatitis, cirrhosis without alcohol), 573 (Other disorders of liver) | B15-B19 | B15-B19 |  |
| **Liver special** | ***NA*** | **789F** | Excluded: 789F (Ascites) | ***NA*** | **R18** | Excluded: R18 (Ascites) |
| **Moderate severe liver disease** | 456A/B/C | 456A/B/C |  | I850/9, I859, I982, I983 | I850/9, I859, I982, I983 |  |
| **Peptic ulcer disease** | ***NA*** | **531-534** | Excluded: 531-534 (Ulcer) | ***NA*** | **K25-K28** | Excluded: K25-K28 (Ulcer) |
| **Malignancy** | **140-189, 200-208** | **140-195, 200-208** | Excluded: 190-195 (Malignant neoplasm: eye, brain, other and unspecified parts of nervous system, thyroid gland, other endocrine glands and related structures, other and ill-defined sites) | **C00-C75, C81-C97** | **C00-C76, C81-C86, C88-C97** | Excluded: C76 ( Malignant neoplasm of other and ill-defined sites) |
| **Metastatic cancer** | **190-199** | **196-198, 199A/B** | Included: 190-195 (Malignant neoplasm: eye, brain, other and unspecified parts of nervous system, thyroid gland, other endocrine glands and related structures, other and ill-defined sites) | **C76-C80** | **C77-C80** | Included: C76 ( Malignant neoplasm of other and ill-defined sites) |
| **Aids** | 279K | **079J**, 279K | Excluded: 079J (Defines causative virus) | B20-B24, F024 | B20-B24, F024, **O987, R75, Z114, Z219, Z717** | Excluded: O0987 (HIV as complication of pregnancy), R75 (Laboratory evidence of HIV), Z114 (Special screening examination for HIV), Z219 (Asymptomatic infection of HIV), Z717 (HIV counselling) |

**Table S4. Demographic baseline data for all subjects with COVID-19 in national registers**

| **Variable** | **Initially**  **hospitalized for COVID-19** | **COVID-19**  **without hospitalization** |
| --- | --- | --- |
| Number | 906,053 | 51,794 |
| Age, mean (SD) | 41.6 (15.0) | 61.0 (15.2) |
| Age, group (%) |  |  |
| 18 to 54 | 716,835 (79.1) | 16,764 (32.4) |
| 55 to 64 | 124,782 (13.8) | 11,568 (22.3) |
| 65 to 74 | 44,346 (4.9) | 11,306 (21.8) |
| 75 to 84 | 20,090 (2.2) | 12,156 (23.5) |
| Sex |  |  |
| Male (%) | 437,878 (48.3) | 31,431 (60.7) |
| Nordic origin* (%) |  |  |
| Yes | 696,877 (76.9) | 34,001 (65.6) |
| No | 206,855 (22.8) | 17,685 (34.1) |
| Need of care (%) |  |  |
| Independent living | 895,046 (98.8) | 46,147 (89.1) |
| Home care | 4,588 (0.5) | 4,098 (7.9) |
| Long-term care facility | 6,419 (0.7) | 1,549 (3.0) |
| Professional position |  |  |
| Other occupation | 317,245 (35.0) | 10,169 (19.6) |
| Blue-collar worker | 125,730 (13.9) | 5,102 (9.9) |
| Early retirement | 10,820 (1.2) | 2,274 (4.4) |
| Essential workers | 122,256 (13.5) | 4,702 (9.1) |
| Hospital staff | 112,902 (12.5) | 3,782 (7.3) |
| Not working | 49,609 (5.5) | 4,323 (8.3) |
| Retired | 29,644 (3.3) | 13,111 (25.3) |
| School staff | 73,859 (8.2) | 1,912 (3.7) |
| Student | 52,981 (5.8) | 772 (1.5) |
| Education^†^ (%) |  |  |
| ≤ 9 years | 131,945 (14.6) | 13,611 (26.3) |
| 10─12 years | 398,623 (44.0) | 22,018 (42.5) |
| College or university | 355,783 (39.3) | 14,361 (27.7) |
| Baseline comorbidities, n (%) |  |  |
| Diabetes | 23,998 (2.6) | 9,064 (17.5) |
| Hypertension | 35282 (3.9) | 14,298 (27.6) |
| Atrial fibrillation | 9,650 (1.1) | 4,525 (8.7) |
| Dementia | 3,821 (0.4) | 1,318 (2.5) |
| COPD | 6,595 (0.7) | 3,865 (7.5) |
| Heart failure | 4,219 (0.5) | 3,424 (6.6) |
| Cancer | 11,143 (1.2) | 2,236 (4.3) |
| Obesity (diagnostic) | 22,694 (2.5) | 2,660 (5.1) |
| Venous thromboembolism | 11,932 (1.3) | 2,933 (5.7) |
| Myocardial infarction | 6,923 (0.8) | 3,148 (6.1) |
| CCI weighted, mean (SD) | 0.75 (1.27) | 2.09 (2.16) |

Abbreviations: COPD: Chronic obstructive pulmonary disease; CCIw: Weighted Charlson Comorbidity Index

*Missing data for Born in Nordic countries were 0.3%

^†^ Missing data for Education were 2.2%

**Table S5. Events and hazard ratios for patients with COVID-19 and pulmonary embolism without concomitant deep venous thrombosis**

|  |  | **Early (0−<60 days)** | | **Late (60−<180 days)** | | **Long-term (from 180 days)** | |
| --- | --- | --- | --- | --- | --- | --- | --- |
| **Age group (years)** | **Cases** | **PE without DVT at index** | **PE without DVT within 60 days** | **PE without DVT late** | **HR (95% CI)**  **Late**  **Model 2^†^** | **PE without DVT long-term** | **HR (95% CI)**  **long-term**  **Model 2^†^** |
| **Hospitalized for COVID-19** |  |  |  |  |  |  |  |
| 18 to 54 | 16,270 | 456 | 67 | 13 | 7.73 (2.92-20.49) | 9 | 2.67 (1.14-6.25) |
| 55 to 64 | 10,979 | 453 | 57 | 55 | 18.84 (10.70-33.19) | 18 | 3.04 (1.68-5.51) |
| 65 to 74 | 10,429 | 422 | 71 | 44 | 5.11 (3.27-7.97) | 14 | 1.40 (0.74-2.64) |
| 75 to 84 | 10,887 | 303 | 77 | 34 | 3.30 (2.11-5.17) | 21 | 1.80 (1.10-2.96) |
| **Total** | 48,565 | **1634** | **272** | **146** | **6.25 (4.88-8.01)** | **62** | **2.05 (1.52-2.76)** |
| **COVID-19 without hospitalization** | | | | | | | |
| 18 to 54 | 710,900 | 7 | 168 | 77 | 1.18 (0.92-1.51) | 67 | 1.15 (0.88-1.50) |
| 55 to 64 | 121,784 | 5 | 91 | 48 | 1.04 (0.76-1.42) | 39 | 0.98 (0.69-1.38) |
| 65 to 74 | 42,451 | 3 | 62 | 42 | 1.69 (1.19-2.40) | 26 | 0.99 (0.65-1.53) |
| 75 to 84 | 18,546 | 1 | 45 | 33 | 1.28 (0.87-1.89) | 28 | 1.38 (0.89-2.12) |
| **Total** | 893,681 | **16** | **366** | **200** | **1.21 (1.04-1.42)** | **160** | **1.09 (0.91-1.29)** |

Abbreviations: PE; pulmonary embolism: DVT; deep venous thrombosis: HR; hazard ratio: CI; 95% confidence interval: CCI; Charlson comorbidity index

**^†^** Model 2 adjusted for age, sex, obesity, hypertension, need of assisted living, born in Nordic countries, education and CCI.

**Table S6.** **Thromboembolic events adjusted for only age and sex in subjects with COVID-19 who were not hospitalized (exposed) and matched individuals without COVID-19 (non-exposed)**

|  |  | **Early (0─<60 days)** | | | **Late (60─<180 days)** | | | | **Long-term (from 180 days)** | | | |
| --- | --- | --- | --- | --- | --- | --- | --- | --- | --- | --- | --- | --- |
| **Age** |  | **N=** | **Events** | **HR (95% CI)**  **Model 1^†^** | **N=*** | **Events** | **Event rate**  **(/1000 years)** | **HR (95% CI)**  **Model 1^†^** | **N=*** | **Events** | **Event rate**  **(/1000 years)** | **HR (95% CI)**  **Model 1^†^** |
| **VTE** |  |  |  |  |  |  |  |  |  |  |  |  |
| 18 to 54 | Exposed | 710,570 | 319 | 3.06 (2.65-3.52) | 710,126 | 206 | 0.92 | 1.09 (0.94-1.27) | 653,695 | 198 | 0.9 | 1.18 (1.01-1.38) |
|  | Non-exposed | 3,550,407 | 502 |  | 3,446,525 | 891 | 0.84 |  | 3,022,616 | 735 | 0.76 |  |
| 54 to 64 | Exposed | 121,640 | 183 | 3.34 (2.75-4.05) | 121,298 | 91 | 2.32 | 0.83 (0.66-1.04) | 116,428 | 93 | 2.24 | 0.98 (0.78-1.23) |
|  | Non-exposed | 608,108 | 260 |  | 593,679 | 500 | 2.65 |  | 550,608 | 431 | 2.3 |  |
| 65 to 74 | Exposed | 42,360 | 126 | 3.79 (2.99-4.80) | 41,715 | 75 | 5.6 | 1.47 (1.13-1.90) | 39,740 | 59 | 4.24 | 1.13 (0.85-1.50) |
|  | Non-exposed | 211,901 | 159 |  | 209,148 | 257 | 3.86 |  | 196,413 | 255 | 3.77 |  |
| 75 to 84 | Exposed | 18,472 | 90 | 4.23 (3.17-5.65) | 16,600 | 57 | 10.88 | 1.39 (1.04-1.85) | 15,457 | 43 | 6.73 | 1.21 (0.87-1.68) |
|  | Non-exposed | 92,609 | 112 |  | 91,367 | 234 | 8.09 |  | 85,665 | 216 | 5.74 |  |
| Total | Exposed | 893,042 | 718 | 3.36 (3.05-3.70) | 889,739 | 429 | 1.52 | 1.10 (0.99-1.22) | 825,320 | 393 | 1.39 | 1.13 (1.01-1.26) |
|  | Non-exposed | 4,463,025 | 1,033 |  | 4,340,719 | 1,882 | 1.4 |  | 3,855,302 | 1,637 | 1.3 |  |
| **PE** |  |  |  |  |  |  |  |  |  |  |  |  |
| 18 to 54 | Exposed | 710,889 | 192 | 3.99 (3.28-4.85) | 710,568 | 83 | 0.37 | 1.10 (0.87-1.40) | 654,205 | 76 | 0.34 | 1.15 (0.90-1.49) |
|  | Non-exposed | 3,552,215 | 227 |  | 3,448,541 | 359 | 0.34 |  | 3,024,882 | 289 | 0.3 |  |
| 54 to 64 | Exposed | 121,777 | 106 | 3.46 (2.67-4.48) | 121,503 | 52 | 1.32 | 0.94 (0.70-1.27) | 116,657 | 44 | 1.06 | 0.94 (0.68-1.30) |
|  | Non-exposed | 608,788 | 143 |  | 594,449 | 262 | 1.39 |  | 551,527 | 212 | 1.13 |  |
| 65 to 74 | Exposed | 42,445 | 82 | 3.88 (2.88-5.23) | 41,834 | 47 | 3.5 | 1.70 (1.22-2.36) | 39,874 | 28 | 2.01 | 0.98 (0.66-1.48) |
|  | Non-exposed | 212,270 | 97 |  | 209,565 | 139 | 2.08 |  | 196,887 | 140 | 2.07 |  |
| 75 to 84 | Exposed | 18,541 | 64 | 4.68 (3.29-6.67) | 16,683 | 37 | 7.02 | 1.40 (0.98-2.01) | 15,550 | 31 | 4.82 | 1.53 (1.03-2.28) |
|  | Non-exposed | 92,804 | 70 |  | 91,594 | 150 | 5.17 |  | 85,936 | 123 | 3.26 |  |
| Total | Exposed | 893,652 | 444 | 3.93 (3.46-4.47) | 890,588 | 219 | 0.77 | 1.18 (1.02-1.37) | 826,286 | 179 | 0.63 | 1.12 (0.95-1.32) |
|  | Non-exposed | 4,466,077 | 537 |  | 4,344,149 | 910 | 0.67 |  | 3,859,232 | 764 | 0.61 |  |
| **DVT** |  |  |  |  |  |  |  |  |  |  |  |  |
| 18 to 54 | Exposed | 710,740 | 141 | 2.21 (1.81-2.70) | 710,465 | 130 | 0.58 | 1.05 (0.87-1.27) | 654,067 | 134 | 0.61 | 1.20 (0.99-1.45) |
|  | Non-exposed | 3,551,362 | 310 |  | 3,447,596 | 582 | 0.55 |  | 3,023,786 | 490 | 0.51 |  |
| 54 to 64 | Exposed | 121,744 | 83 | 2.90 (2.20-3.82) | 121,494 | 44 | 1.12 | 0.69 (0.50-0.97) | 116,657 | 58 | 1.4 | 1.03 (0.78-1.37) |
|  | Non-exposed | 608,648 | 139 |  | 594,297 | 278 | 1.47 |  | 551,337 | 256 | 1.37 |  |
| 65 to 74 | Exposed | 42,444 | 50 | 3.42 (2.38-4.92) | 41,851 | 30 | 2.23 | 1.12 (0.75-1.66) | 39,900 | 35 | 2.5 | 1.30 (0.89-1.88) |
|  | Non-exposed | 212,233 | 73 |  | 209,538 | 135 | 2.02 |  | 196,855 | 131 | 1.93 |  |
| 75 to 84 | Exposed | 18,545 | 31 | 3.56 (2.23-5.69) | 16,696 | 23 | 4.36 | 1.44 (0.91-2.27) | 15,566 | 15 | 2.33 | 0.85 (0.49-1.46) |
|  | Non-exposed | 92,835 | 47 |  | 91,629 | 91 | 3.13 |  | 85,993 | 106 | 2.81 |  |
| Total | Exposed | 893,473 | 305 | 2.63 (2.29-3.03) | 890,506 | 227 | 0.8 | 1.00 (0.86-1.15) | 826,190 | 242 | 0.86 | 1.15 (1.00-1.32) |
|  | Non-exposed | 4,465,078 | 569 |  | 4,343,060 | 1086 | 0.8 |  | 3,857,971 | 983 | 0.78 |  |

**Table S6. Continued**

^*^ Cases/matched non-exposed controls that were alive, had not experienced an event and were still in follow-up.

^†^ Model 1 is adjusted for age and sex only.

Abbreviations: N; number: HR; hazard ratio: CI; 95% confidence interval: VTE; venous thromboembolism: PE; pulmonary embolism: DVT; deep venous thrombosis.

**Table S7. Thromboembolic events in patients hospitalized for COVID-19 (exposed) and subjects without COVID-19 (non-exposed) with CCI=0**

|  |  | **Early (0─<60 days)** | | | | **Late (60─<180 days)** | | | | **Long-term (from 180 days)** | | | |
| --- | --- | --- | --- | --- | --- | --- | --- | --- | --- | --- | --- | --- | --- |
| **Age** | **COVID-19 status** | **N=** | **During initial hospitalization** | **After hospitalization*** | **Total** | **N=†** | **Events** | **Event rate (/1000 years)** | **HR (CI)**  **Model 1^‡^** | **N=†** | **Events** | **Event rate (/1000 years)** | **HR (CI)**  **Model 1^‡^** |
| **VTE** |  |  |  |  |  |  |  |  |  |  |  |  |  |
| 18 to 54 | Exposed | 9,658 | 331 | 61 | 392 | 9,183 | 21 | 7.26 | 9.53 (4.23-21.46) | 8,239 | 8 | 2.0 | 1.75 (0.75-4.10) |
|  | Non-exposed | 36,149 |  | 4 | 4 | 35,118 | 8 | 0.73 |  | 30,351 | 16 | 1.16 |  |
| 55 to 64 | Exposed | 5,120 | 288 | 32 | 320 | 4,661 | 37 | 24.78 | 14.95 (6.95-32.15) | 4,371 | 7 | 3.28 | 2.13 (0.81-5.60) |
|  | Non-exposed | 16,152 |  | 7 | 7 | 15,343 | 8 | 1.64 |  | 14,087 | 11 | 1.71 |  |
| 65 to 74 | Exposed | 2,969 | 217 | 33 | 250 | 2,451 | 14 | 17.8 | 8.56 (2.82-25.97) | 2,334 | 4 | 3.65 | 1.99 (0.54-7.39) |
|  | Non-exposed | 7,165 |  | 8 | 8 | 6,341 | 4 | 1.95 |  | 5,999 | 5 | 1.83 |  |
| 75 to 84 | Exposed | 1,742 | 116 | 19 | 135 | 1,279 | 12 | 29.44 | 3.87 (1.40-10.71) | 1,215 | 8 | 14.12 | 3.05 (0.9-10.28) |
|  | Non-exposed | 2,714 |  | 1 | 1 | 2,151 | 5 | 7.2 |  | 2,028 | 4 | 4.49 |  |
| Total | Exposed | 19,489 | 952 | 145 | 1,097 | 17,574 | 84 | 15.05 | 9.70 (6.19-15.22) | 16,159 | 27 | 3.46 | 2.16 (1.30-3.57) |
|  | Non-exposed | 62,180 |  | 20 | 20 | 58,953 | 25 | 1.35 |  | 52,465 | 36 | 1.51 |  |
| **PE** |  |  |  |  |  |  |  |  |  |  |  |  |  |
| 18 to 54 | Exposed | 9,667 | 307 | 40 | 347 | 9,237 | 12 | 4.12 | 11.15 (3.61-34.42) | 8,296 | 3 | 0.74 | 1.51 (0.39-5.88) |
|  | Non-exposed | 36,160 |  | 1 | 1 | 35,132 | 4 | 0.37 |  | 30,365 | 7 | 0.51 |  |
| 55 to 64 | Exposed | 5,126 | 273 | 26 | 299 | 4,687 | 34 | 22.64 | 18.23 (7.65-43.40) | 4,399 | 5 | 2.32 | 3.00 (0.87-0.35) |
|  | Non-exposed | 16,164 |  | 1 | 1 | 15,360 | 6 | 1.23 |  | 14,104 | 6 | 0.93 |  |
| 65 to 74 | Exposed | 2,980 | 211 | 28 | 239 | 2,468 | 11 | 13.88 | 9.08 (2.53-32.54) | 2,353 | 4 | 3.61 | 3.31 (0.75-4.61) |
|  | Non-exposed | 7,177 |  | 3 | 3 | 6,356 | 3 | 1.46 |  | 6,014 | 3 | 1.09 |  |
| 75 to 84 | Exposed | 1,749 | 110 | 18 | 128 | 1,289 | 12 | 29.21 | 9.24 (2.08-40.95) | 1,225 | 6 | 10.47 | 2.88 (0.71-1.72) |
|  | Non-exposed | 2,723 |  | 1 | 1 | 2,156 | 2 | 2.87 |  | 2,033 | 3 | 3.36 |  |
| Total | Exposed | 19,522 | 901 | 112 | 1,013 | 17,681 | 69 | 12.28 | 12.97 (7.40-22.74) | 16,273 | 18 | 2.29 | 2.72 (1.41-5.22) |
|  | Non-exposed | 62,224 |  | 6 | 6 | 59,004 | 15 | 0.81 |  | 52,516 | 19 | 0.79 |  |
| **DVT** |  |  |  |  |  |  |  |  |  |  |  |  |  |
| 18 to 54 | Exposed | 9,698 | 33 | 27 | 60 | 9,541 | 14 | 4.65 | 12.05 (3.98-36.55) | 8,562 | 5 | 1.21 | 1.86 (0.62-5.58) |
|  | Non-exposed | 36,155 |  | 3 | 3 | 35,125 | 4 | 0.37 |  | 30,360 | 9 | 0.65 |  |
| 55 to 64 | Exposed | 5,139 | 24 | 10 | 34 | 4,937 | 6 | 3.78 | 6.05 (1.51-24.31) | 4,658 | 2 | 0.88 | 0.81 (0.17-3.97) |
|  | Non-exposed | 16,168 |  | 6 | 6 | 15,356 | 3 | 0.61 |  | 14,100 | 7 | 1.09 |  |
| 65 to 74 | Exposed | 2,995 | 10 | 9 | 19 | 2,651 | 3 | 3.52 | 6.35 (0.68-59.26) | 2,534 | 1 | 0.84 | 0.74 (0.08-7.08) |
|  | Non-exposed | 7,173 |  | 5 | 5 | 6,347 | 1 | 0.49 |  | 6,007 | 3 | 1.1 |  |
| 75 to 84 | Exposed | 1,752 | 7 | 5 | 12 | 1,381 | 1 | 2.26 | 0.56 (0.06-5.29) | 1,314 | 4 | 6.55 | 6.34 (0.69-8.20) |
|  | Non-exposed | 2,720 |  | 0 | 0 | 2,157 | 3 | 4.31 |  | 2,034 | 1 | 1.2 |  |
| Total | Exposed | 19,584 | 74 | 51 | 125 | 18,510 | 24 | 4.07 | 6.19 (2.99-12.78) | 17,068 | 12 | 1.46 | 1.66 (0.81-3.40) |
|  | Non-exposed | 62,216 |  | 14 | 14 | 58,985 | 11 | 0.59 |  | 52,501 | 20 | 0.84 |  |

^*^  Refers to events occurring after discharge from first hospitalization. The non-exposed groups include all events occurring during the full 60-day period.

^†^ Number that were alive, had not experienced an event and were still in follow-up.

^‡^ Model 1 adjusted for age and sex.

Abbreviations: CCI; Charlson Comorbidity Index: N; number: HR; hazard ratio: CI; 95% confidence interval: VTE; venous thromboembolism: PE; pulmonary embolism: DVT; deep venous thrombosis.

**Table S8. Thromboembolic events in subjects with COVID-19 without hospitalization (exposed) and without COVID-19 (non-exposed) with CCI=0**

|  |  | **Early (0─<60 days )** | | | **Late (60─<180 days)** | | | | **Long-term (from 180 days)** | | | |
| --- | --- | --- | --- | --- | --- | --- | --- | --- | --- | --- | --- | --- |
| **Age** | **COVID-19 status** | **N=** | **Events** | **HR (CI)**  **Model 1†** | **N=*** | **Events** | **Event rate**  **(/1000 years)** | **HR (CI)**  **Model 1†** | **N=*** | **Events** | **Event rate**  **(/1000 years)** | **HR (CI)**  **Model 1†** |
| **VTE** |  |  |  |  |  |  |  |  |  |  |  |  |
| 18 to 54 | Exposed | 523,057 | 219 | 3.7 (3.06-4.48) | 522,781 | 123 | 0.74 | 1.17 (0.95-1.44) | 480,417 | 114 | 0.71 | 1.15 (0.93-1.43) |
|  | Non-exposed | 1,942,298 | 208 |  | 1,886,121 | 364 | 0.63 |  | 1,651,304 | 319 | 0.61 |  |
| 54 to 64 | Exposed | 77,149 | 111 | 4.85 (3.57-6.6) | 76,985 | 56 | 2.25 | 1.00 (0.73-1.36) | 73,941 | 42 | 1.61 | 1.02 (0.72-1.45) |
|  | Non-exposed | 241,924 | 68 |  | 236,024 | 162 | 2.16 |  | 218,891 | 116 | 1.58 |  |
| 65 to 74 | Exposed | 19,757 | 53 | 3.59 (2.31-5.57) | 19,625 | 29 | 4.6 | 1.57 (0.98-2.51) | 18,743 | 18 | 2.84 | 0.87 (0.50-1.50) |
|  | Non-exposed | 48,902 | 33 |  | 48,082 | 44 | 2.87 |  | 45,119 | 47 | 3.19 |  |
| 75 to 84 | Exposed | 4,488 | 20 | 4.23 (1.77-10.07) | 4,309 | 14 | 10.25 | 1.61 (0.77-3.36) | 4,036 | 7 | 4.71 | 1.31 (0.48-3.59) |
|  | Non-exposed | 7,313 | 7 |  | 7,042 | 14 | 6.3 |  | 6,466 | 8 | 3.47 |  |
| Total | Exposed | 624,451 | 403 | 3.98 (3.43-4.62) | 623,700 | 222 | 1.12 | 1.19 (1.02-1.39) | 577,137 | 181 | 0.93 | 1.08 (0.91-1.28) |
|  | Non-exposed | 2,240,437 | 316 |  | 2,177,269 | 584 | 0.87 |  | 1,921,780 | 490 | 0.80 |  |
| **PE** |  |  |  |  |  |  |  |  |  |  |  |  |
| 18 to 54 | Exposed | 523,228 | 139 | 5.54 (4.22-7.26) | 523,028 | 40 | 0.24 | 0.97 (0.68-1.38) | 480,716 | 43 | 0.27 | 1.12 (0.79-1.59) |
|  | Non-exposed | 1,943,009 | 88 |  | 1,886,933 | 144 | 0.25 |  | 1,652,221 | 125 | 0.24 |  |
| 54 to 64 | Exposed | 77,220 | 61 | 4.95 (3.24-7.58) | 77,103 | 31 | 1.24 | 1.06 (0.71-1.60) | 74,077 | 19 | 0.73 | 1.02 (0.60-1.72) |
|  | Non-exposed | 242,151 | 35 |  | 236,276 | 87 | 1.16 |  | 219,193 | 52 | 0.71 |  |
| 65 to 74 | Exposed | 19,786 | 38 | 4.08 (2.35-7.06) | 19,667 | 20 | 3.17 | 1.97 (1.09-3.58) | 18,793 | 6 | 0.94 | 0.62 (0.25-1.52) |
|  | Non-exposed | 48,980 | 20 |  | 48,170 | 24 | 1.56 |  | 45,216 | 22 | 1.49 |  |
| 75 to 84 | Exposed | 4,504 | 16 | 5.79 (1.93-17.39) | 4,326 | 8 | 5.83 | 2.54 (0.84-7.70) | 4,057 | 6 | 4.02 | 1.47 (0.48-4.51) |
|  | Non-exposed | 7,325 | 4 |  | 7,057 | 5 | 2.24 |  | 6,486 | 6 | 2.59 |  |
| Total | Exposed | 624,738 | 254 | 5.21 (4.24-6.41) | 624,124 | 99 | 0.50 | 1.19 (0.94-1.50) | 577,643 | 74 | 0.38 | 1.05 (0.80-1.37) |
|  | Non-exposed | 2,241,465 | 147 |  | 2,178,436 | 260 | 0.39 |  | 1,923,116 | 205 | 0.33 |  |
| **DVT** |  |  |  |  |  |  |  |  |  |  |  |  |
| 18 to 54 | Exposed | 523,145 | 91 | 2.38 (1.83-3.11) | 522,991 | 86 | 0.52 | 1.25 (0.98-1.61) | 480,641 | 80 | 0.50 | 1.20 (0.92-1.55) |
|  | Non-exposed | 1,942,653 | 135 |  | 1,886,523 | 238 | 0.41 |  | 1,651,757 | 214 | 0.41 |  |
| 54 to 64 | Exposed | 77,202 | 52 | 4.42 (2.87-6.81) | 77,094 | 29 | 1.16 | 0.86 (0.56-1.32) | 74,074 | 28 | 1.07 | 1.03 (0.67-1.59) |
|  | Non-exposed | 242,062 | 37 |  | 236,186 | 94 | 1.25 |  | 219,091 | 78 | 1.06 |  |
| 65 to 74 | Exposed | 19,780 | 17 | 2.6 (1.29-5.26) | 19,674 | 9 | 1.42 | 0.94 (0.43-2.03) | 18,806 | 13 | 2.04 | 1.02 (0.53-1.96) |
|  | Non-exposed | 48,954 | 15 |  | 48,147 | 23 | 1.50 |  | 45,191 | 29 | 1.97 |  |
| 75 to 84 | Exposed | 4,500 | 5 | 2.16 (0.49-9.61) | 4,333 | 6 | 4.37 | 1.11 (0.40-3.07) | 4,065 | 3 | 2.00 | 2.31 (0.39-13.82) |
|  | Non-exposed | 7,331 | 3 |  | 7,062 | 9 | 4.03 |  | 6,488 | 2 | 0.86 |  |
| Total | Exposed | 624,627 | 165 | 2.78 (2.26-3.44) | 624,092 | 130 | 0.66 | 1.12 (0.91-1.37) | 577,586 | 124 | 0.64 | 1.13 (0.92-1.40) |
|  | Non-exposed | 2,241,000 | 190 |  | 2,177,918 | 364 | 0.54 |  | 1,922,527 | 323 | 0.53 |  |

^*^ Numbers that were alive, had not experienced an event and were still in follow-up.

^†^ Model 1 adjusted for age and sex.

Abbreviations: CCI; Charlson Comorbidity Index: N; number: HR; hazard ratio: CI; 95% confidence interval: VTE; venous thromboembolism: PE; pulmonary embolism: DVT; deep venous thrombosis.

**Table S9. Death, events and hazards, for patients who were initially hospitalized for COVID-19 and individuals with COVID-19 without hospitalization (exposed) and matched controls without COVID-19 (non-exposed).**

| **COVID-19 status whole**  **cohort** | **N=** | **Early (0─<60 days)** | | **Late (60─<180 days)** | | | **Long-term (from 180 days)** | | |
| --- | --- | --- | --- | --- | --- | --- | --- | --- | --- |
|  |  | **Events** | **HR (95% CI)**  **Model 1^†^** | **Events** | **Events**  **/1000**  **years** | **HR (95% CI)**  **Model 1^†^** | **Events** | **Events**  **/1000**  **years** | **HR (95% CI)**  **Model 1^†^** |
| **Hospitaliztion for COVID-19** | | | | | | | | | |
| Exposed | 48,861 | 5518 | 63.5 (57.93-69.61) | 661 | 47,87 | 4.73 (4.28-5.23) | 608 | 30,75 | 2.79 (2.54-3.07) |
| Non-exposed | 244,242 | 509 |  | 912 | 11,94 |  | 1458 | 13,67 |  |
| **COVID-19 without hospitalization** | | | | | | | | | |
| Exposed | 894,121 | 2706 | 8.28 (7.78-8.82) | 801 | 2,83 | 1.22 (1.13-1.32) | 1007 | 3,56 | 1.40 (1.31-1.50) |
| Non-exposed | 4,468,411 | 1690 |  | 3337 | 2,47 |  | 3694 | 2,94 |  |

^†^ Model 1 adjusted for age and sex.

Abbreviations: N; number: HR; hazard ratio: CI; 95% confidence intervals

**Figure S1. Detailed inclusion exclusion chart, including procedures for weekly matching**


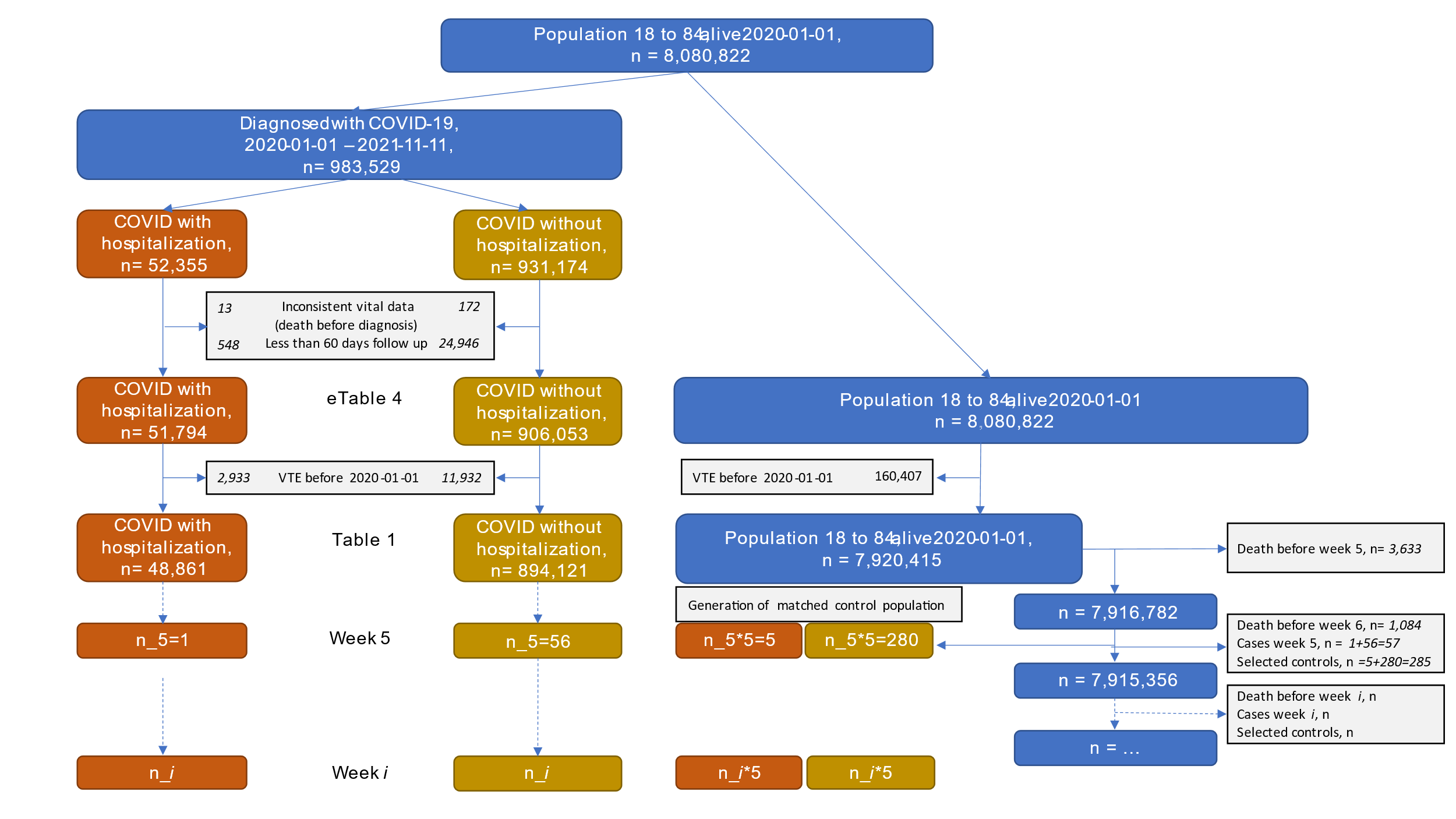


Generation of matched control population: For each consecutive week, initially hospitalized and non-hospitalized patients were matched by age and sex to five individuals from the control-pool. The control-pool was updated by removing the selected controls and all cases. This was repeated for consecutive weeks.

Abbreviations: VTE; venous thromboembolism: n_*i;* the number of cases per week (*i)* (which was matched to 5 controls/case)

**Figure S2.** Cumulative incidence for VTE with competing risk for death by age groups in patients initially hospitalized for COVID-19 (exposed) and population-derived subjects with no COVID-19 (non-exposed)

**
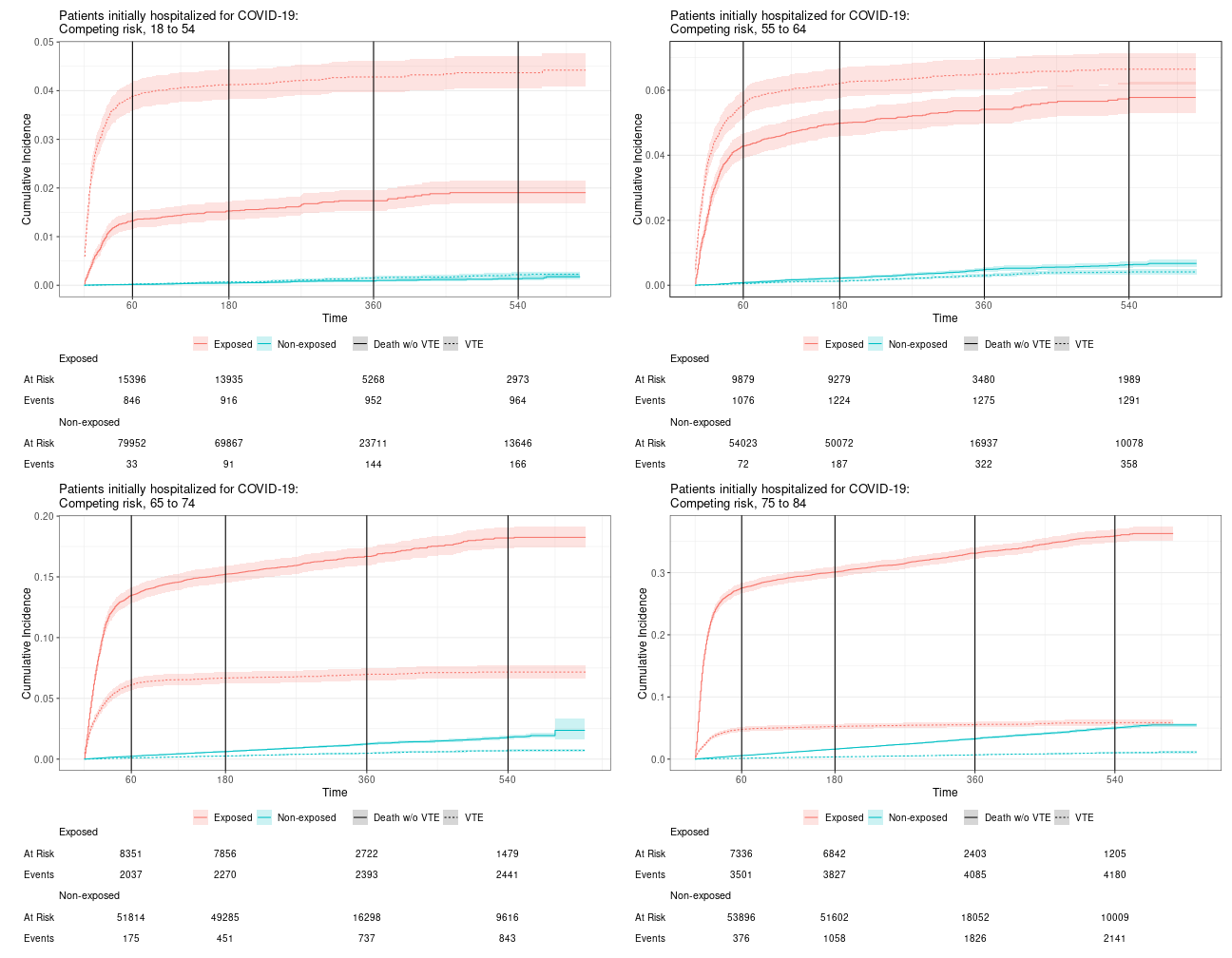
**

Abbreviations: VTE; venous thromboembolism: w/o; without

**Figure S3.** Cumulative incidence for VTE and death without VTE by age groups in subjects with COVID-19 without hospitalization (exposed) and population-derived subjects with no COVID-19 (non-exposed)


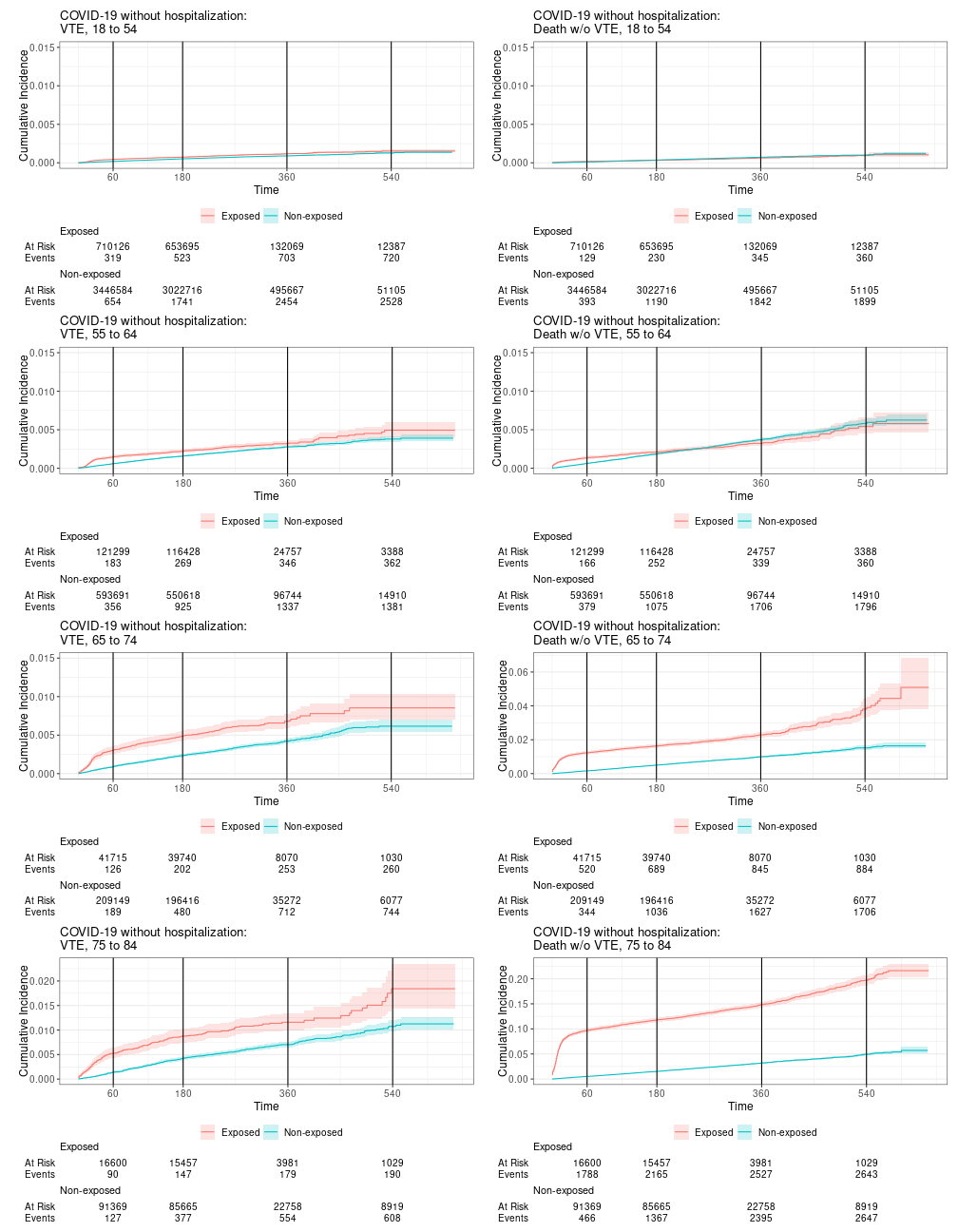


Abbreviations: VTE; venous thromboembolism: w/o; without

**Figure S4.** Cumulative incidence for PE with competing risk for death by age groups in patients initially hospitalized for COVID-19 (exposed) and population-derived subjects with no COVID-19 (non-exposed)

**
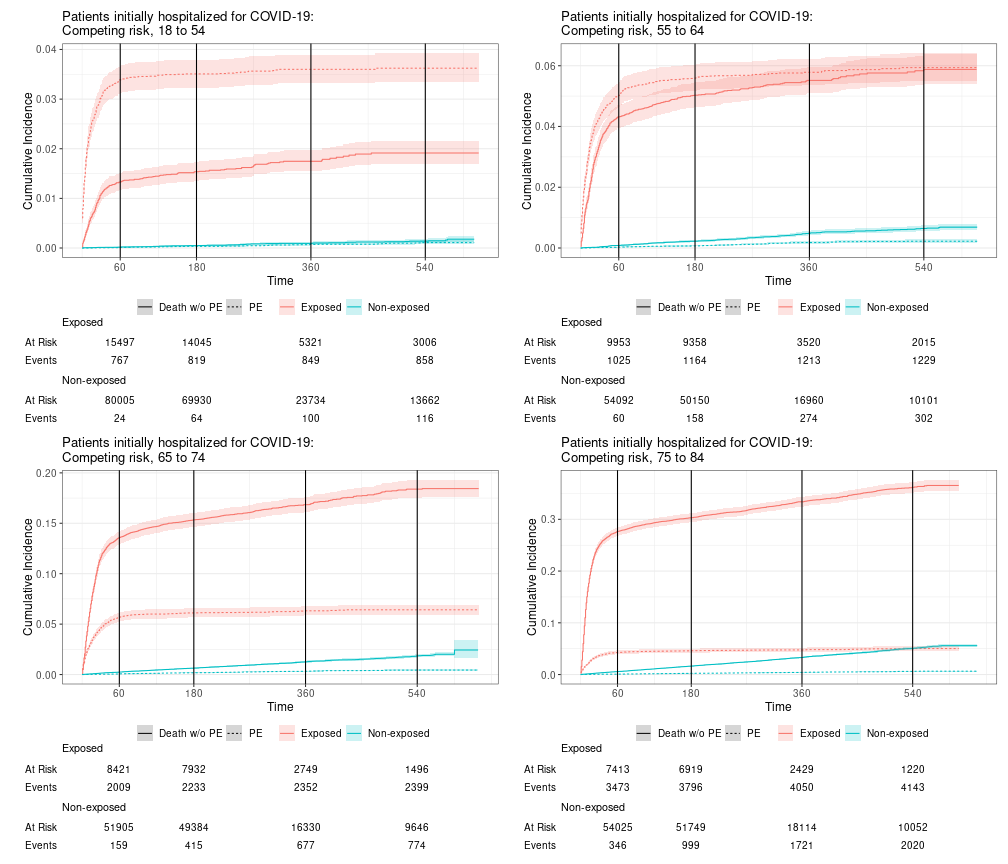
**

Abbreviations: PE; pulmonary embolism: w/o; without

**Figure S5**. Cumulative incidence for PE and death without PE by age groups in subjects with COVID-19 without hospitalization (exposed) and population-derived subjects with no COVID-19 (non-exposed)


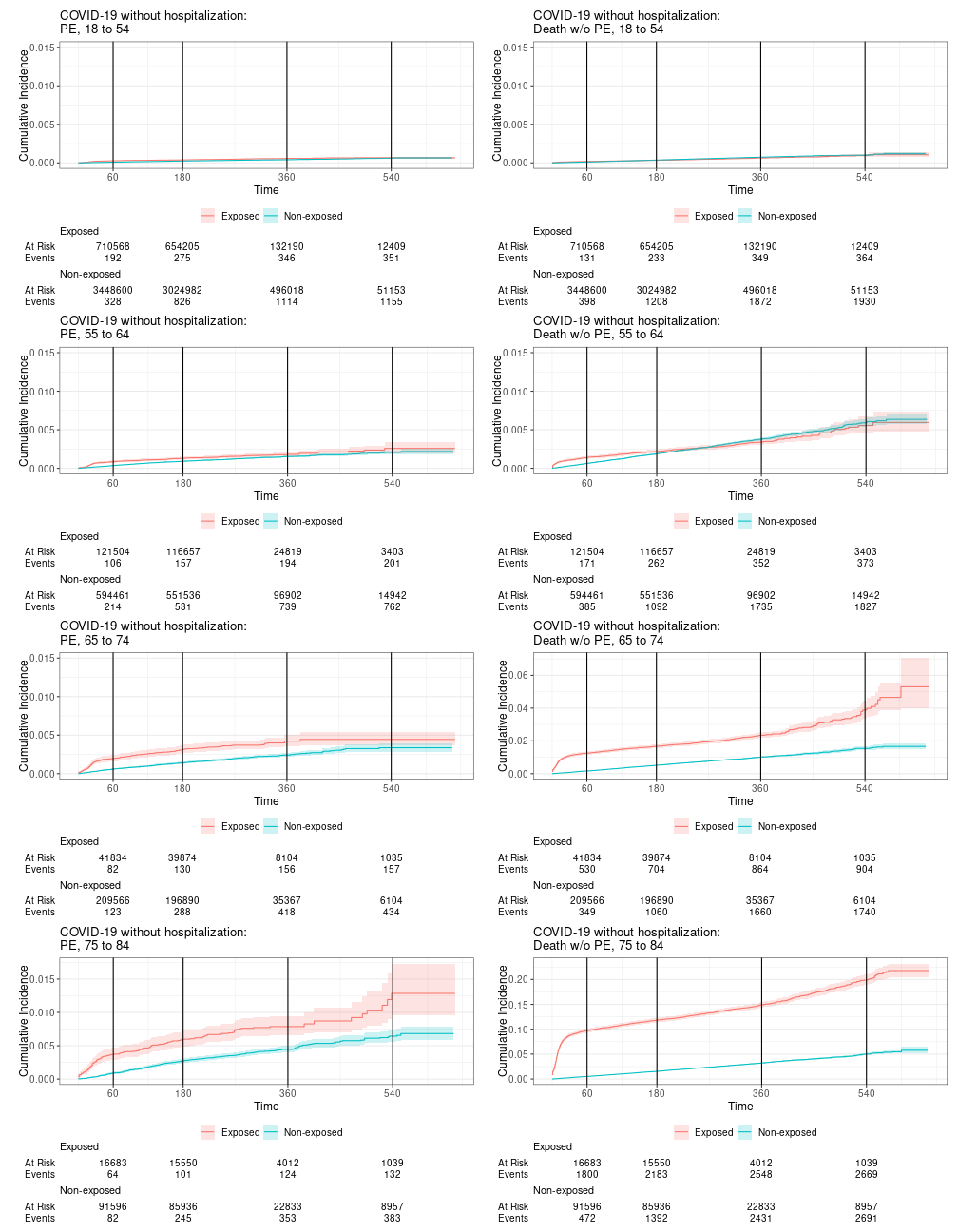


Abbreviations: PE; pulmonary embolism: w/o; without

**Figure S6.** Cumulative incidence for DVT with competing risk for death by age groups in patients initially hospitalized for COVID-19 (exposed) and population-derived subjects with no COVID-19 (non-exposed)

**
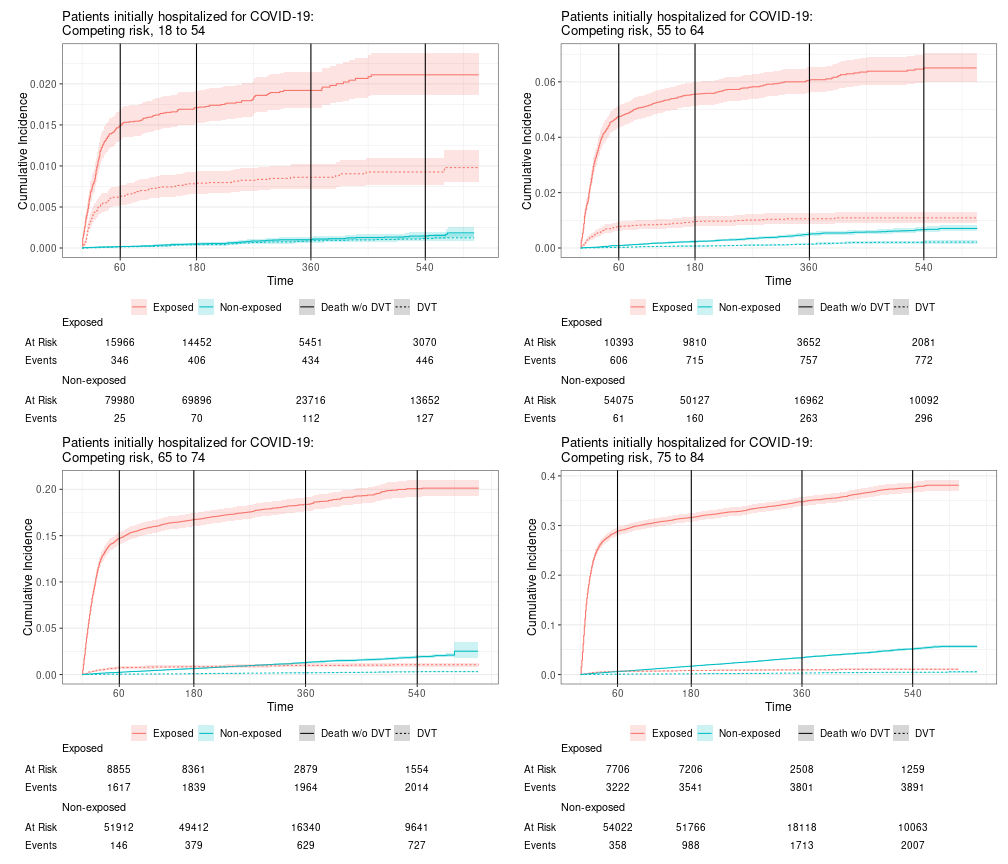
**

Abbreviations: DVT; deep venous thrombosis: w/o; without

**Figure S7.** Cumulative incidence for DVT and death without DVT by age groups in subjects with COVID-19 without hospitalization (exposed) and population-derived subjects with no COVID-19 (non-exposed)


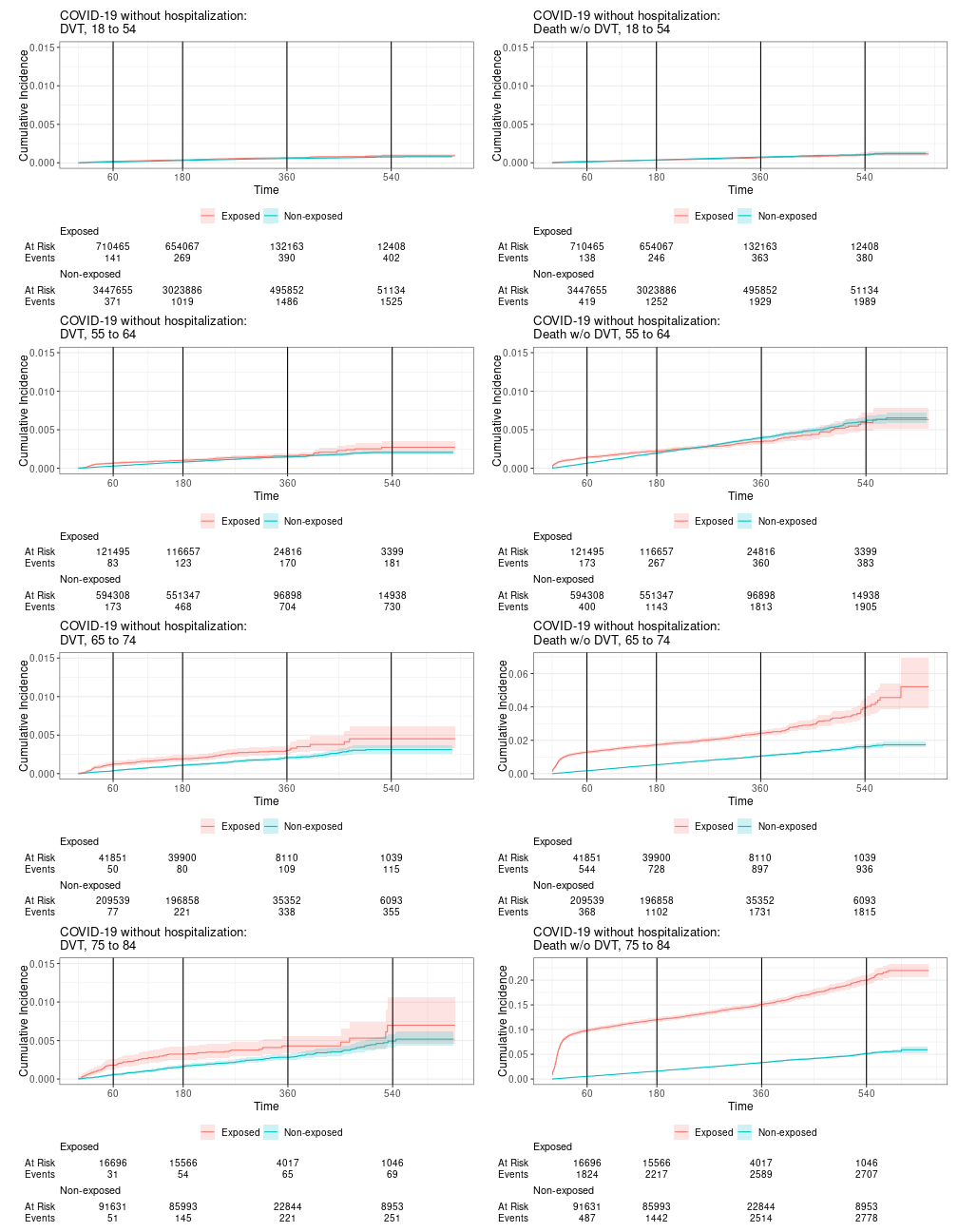
Abbreviations: DVT; deep venous thrombosis: w/o; without
